# Woodlands Facilitate Reproductive Behaviour and Niche Partitioning in Farmland Bumblebee Communities

**DOI:** 10.1002/ece3.73415

**Published:** 2026-04-13

**Authors:** Guthrie Allen, Lynn V. Dicks, Martin I. Taylor, Daniel Hewitt, Richard G. Davies

**Affiliations:** ^1^ School of Biological Sciences University of East Anglia, Norwich Research Park Norwich UK; ^2^ Department of Zoology University of Cambridge Cambridge UK; ^3^ The Woodland Trust Grantham, Lincolnshire UK

**Keywords:** canopy, floral resources, heatwaves, light, microclimate, pollinators, temperate agricultural landscape, understory

## Abstract

Open and closed woodland likely dominated Europe's landscapes during the evolution of its contemporary pollinator diversity, contributing distinct floral and non‐floral resources and cooler and darker microclimates to otherwise treeless environments. As such, we would expect present‐day, generalist pollinator species, such as farmland bumblebees, to exploit woodland resources to differing degrees, and for evolutionary adaptations, such as species' light sensitivity, to accord with relative use of shaded understories. We would also expect exploitation to differ between the pollen‐collecting and reproductive castes. However, a lack of temperate woodland sampling has meant these predictions remain largely untested. To address this knowledge gap, we sampled bumblebee activity (activity‐density) with blue vane traps in shaded woodland understories and in sun‐exposed field margins and woodland canopies from May to July, at 12 sites across agricultural landscapes in Norfolk, England. We then examined relative levels of activity in the understory according to bumblebee species and their light sensitivity, and bumblebee caste. We found that levels of activity in the understory were highly species‐specific and greatest for *Bombus hortorum* L. and *B. pratorum* L. Across species, activity levels in the two sun‐exposed habitats (relative to the understory) were highly positively correlated with each other, and the most light‐sensitive species were more active in the understory. Additionally, the reproductive castes were more active in the understory than workers, especially when temperatures reached heatwave thresholds. These results suggest that woodlands (1) support bumblebee reproductive behaviour, (2) have played a key role in facilitating niche differentiation, mediated by the provision of shade and (3) could be important for maintaining bumblebee diversity in agricultural landscapes—and under a warming climate—with attendant benefits for pollination services.

## Graphical abstract

We sampled farmland bumblebees from habitats largely neglected in European pollinator studies: woodland understories and canopies. Light‐sensitive species and the reproductive castes showed the highest relative activity in shaded woodland understories, with reproductives increasing their use of this habitat during the hottest weather. Our results suggest that woodlands support bumblebee reproductive behaviour, have played a key role in facilitating niche differentiation, and could be important for maintaining bumblebee diversity in agricultural landscapes—and under a warming climate—with attendant benefits for pollination services.

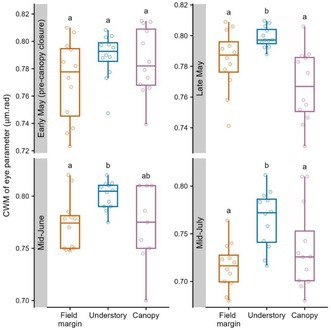

## 1 Introduction

In the prehistoric landscapes of Europe, closed and open woodlands/forests were a dominant feature and likely integral to the evolution of much contemporary European biodiversity (Pearce et al. 2023). Within open landscapes, woodlands contribute unique resources that may be exploited by mobile organisms such as insect pollinators that are nonetheless mostly active in open habitats (Ulyshen et al. 2023). These include resources provided by the trees themselves (e.g., tree holes and leaf litter), distinct floral communities and darker and cooler microhabitats. Woodlands, therefore, increase landscape heterogeneity and the potential for niche partitioning both within and among pollinator species (De Frenne et al. 2021). At shorter timescales, shaded woodland understories may also provide refuge to mobile ectotherms during heat waves, which are increasing in frequency and severity (Woods et al. 2015; Carter et al. 2025). A focus on woodlands, therefore, can shed light on the co‐existence and persistence of pollinating insect species in present‐day ecosystems.

Bumblebees are among the most important crop pollinators in northern temperate regions (Goulson 2010), but to persist in agricultural landscapes, they require semi‐natural habitats that provide season‐long provision of food, nesting and dormancy sites (Cole et al. 2020) and possibly the micro‐site features used for locating mates (Fussell and Corbet 1992). Though previously an understudied habitat, there is now growing evidence that woodlands can benefit wild bee communities in temperate regions (reviewed in Ulyshen et al. 2023), and some bumblebee species and castes are positively associated with this habitat (Svensson et al. 2000; O'Connor et al. 2017; Pfeiffer et al. 2019; Sõber et al. 2020; Maurer et al. 2022; Whitehorn et al. 2022; Tichit et al. 2024).

Woodlands have unique early‐season floral communities that may differentially attract certain species and castes, particularly those with longer tongues well‐suited for feeding on tubular flowers (Allen et al. 2025), and species abundant in spring. Beyond foraging, woodlands may support bumblebee reproduction through the provision of nesting sites (e.g., underground cavities, leaf litter, fallen logs) (Mola et al. 2021) and micro‐site features suitable for scent marking and patrolling males (e.g., tree trunks, branch leaves) (Bringer 1973). We may, therefore, expect the reproductive castes—queens and males—to be relatively more active than the pollen‐collecting workers in woodland understories as the former perform nest‐searching and patrolling behaviours while the latter do not. Additionally, the potential nesting and patrolling microhabitats of woodlands differ from those provided in open habitats and may be differentially favoured among species (Bringer 1973; Fussell and Corbet 1992; Svensson et al. 2000; Kells and Goulson 2003; Pugesek et al. 2024). Finally, the darker and cooler microclimates of woodland understories may be better tolerated by certain species (Bartholomée et al. 2023; Feuerborn et al. 2023; Tichit et al. 2024) or castes, and during heatwaves, they may offer refuge to woodland‐associated and/or heat‐sensitive members of the bumblebee community (Allen et al. 2025).

Recent studies have begun to reveal the relative strengths of woodland association among the common European bumblebee species. It has been found that *Bombus pascuorum* scop., *B. pratorum* L. and *B. hortorum* L. are generally most strongly associated with this habitat, and *B. terrestris* L. and *B. lapidarius* L. are most weakly (Svensson et al. 2000; Kreyer et al. 2004; Allen and Davies 2023; Whitehorn et al. 2022; Tichit et al. 2024). However, few studies have explicitly sampled woodland interiors (e.g., Svensson et al. 2000; Kreyer et al. 2004; Allen and Davies 2023) or compared all five common species (e.g., Allen and Davies 2023; Tichit et al. 2024), and none have combined these attributes and additionally distinguished between spring and summer sampling. Moreover, to our knowledge, only a single study has compared woodland associations between the pollen‐collecting and reproductive castes (Whitehorn et al. 2022). As such, we still have a limited understanding of how the common European species, and their castes, exploit this habitat across seasons, and how woodlands support niche partitioning both within and between species.

Recently, the ‘eye parameter’ (μm.rad), which differs among—but is consistent within—species, was determined as a useful morphological predictor of woodland association and shade tolerance. This measure is the product of the facet diameter (μm) and the angle between two adjacent facets (radian) and represents an evolved trade‐off between light sensitivity and visual resolution, varying independently of species' body and eye size (Bartholomée et al. 2023). Higher values indicate greater investment in light sensitivity and, thus, increased visual performance at low light levels. Bartholomée et al. (2023) show that light‐sensitive species are more active under darker conditions in hemi‐boreal forest, while Tichit et al. (2024) show that the community weighted mean of the eye parameter of grassland bumblebee communities increases with surrounding forest cover. However, it is yet to be determined if a species' eye parameter is related to its relative use of distinct shaded and sun‐exposed habitats, provided, for example, by woodland understories, and canopies and field margins, respectively. If it is, we would expect the community weighted mean of the eye parameter to be highest in the understory. We would also expect activity levels in the two sun‐exposed habitats, across species and relative to the understory, to be positively correlated with each other, such that species with higher field margin activity are also more likely to be found in canopies.

Additionally, larger bumblebee individuals may be more likely to enter woodland habitats. Following Bergmann's rule, larger individuals should be better at retaining heat in cooler conditions and poorer at losing heat in warmer conditions. Larger individuals have bigger eyes (increasing their light sensitivity) and longer tongues (increasing their ability to feed on tubular flowers) and are able to travel greater distances (Fitzgerald et al. 2022). However, the effects of size variation on habitat use are confounded by species and caste identity. It is informative, therefore, to examine the considerable size variation among the foraging workers of each species. There is evidence that worker size variation impacts foraging activity (Peat, Tucker, and Goulson 2005; Ohashi et al. 2008; Hall et al. 2021); however, to our knowledge, there have been no studies that have investigated its effects on habitat use. Such information could add to the understanding of why such size variation exists among the foraging workers of a single colony: it may aid the exploitation of distinct habitats in the local landscape.

To investigate woodland exploitation by—and related niche partitioning within and between –bumblebee species in farmed landscapes, we took a novel approach, sampling bees in shaded woodland understories, and in sun‐exposed field margins and woodland canopies, from late spring to mid‐summer (before and after canopy closure). Sampling was achieved with blue vane traps, and we use the term ‘activity’ to describe trap catches, as counts reflect the local abundance and movements of individuals (activity‐density), rather than true population size. We explored woodland associations in relation to bumblebee species and their eye parameter, bumblebee caste and worker body size, enabling us to address the following questions:

*Do relative activity levels in the woodland understory differ among the common bumblebee species, and are patterns consistent across the seasons*?

*Across species, are activity levels in the two sun‐exposed habitats (field margin and canopy, relative to the understory) positively correlated*?

*Are species with more light‐sensitive eyes (higher eye parameters) more active in the understory*?

*Are the reproductive castes more active in the understory than workers, and if so, which species are driving this trend*?

*Within species, are larger workers more likely to enter woodland habitats*?

Additionally, we examine the effect on levels of understory activity of temperatures that met heatwave thresholds (Met Office 2025) during one of the sampling periods. We consider the potential mechanisms that best explain our results and what these indicate about the role of woodlands in supporting bumblebee reproduction and niche partitioning. We then highlight the implications for conserving bumblebee diversity and promoting pollination services in farmed landscapes under a warming climate.

## 2 Materials and Methods

The sampling design and field work of the present study are as described in Allen et al. (2025), but key aspects are repeated here.

### 2.1 Sampling Sites

**FIGURE 1:**
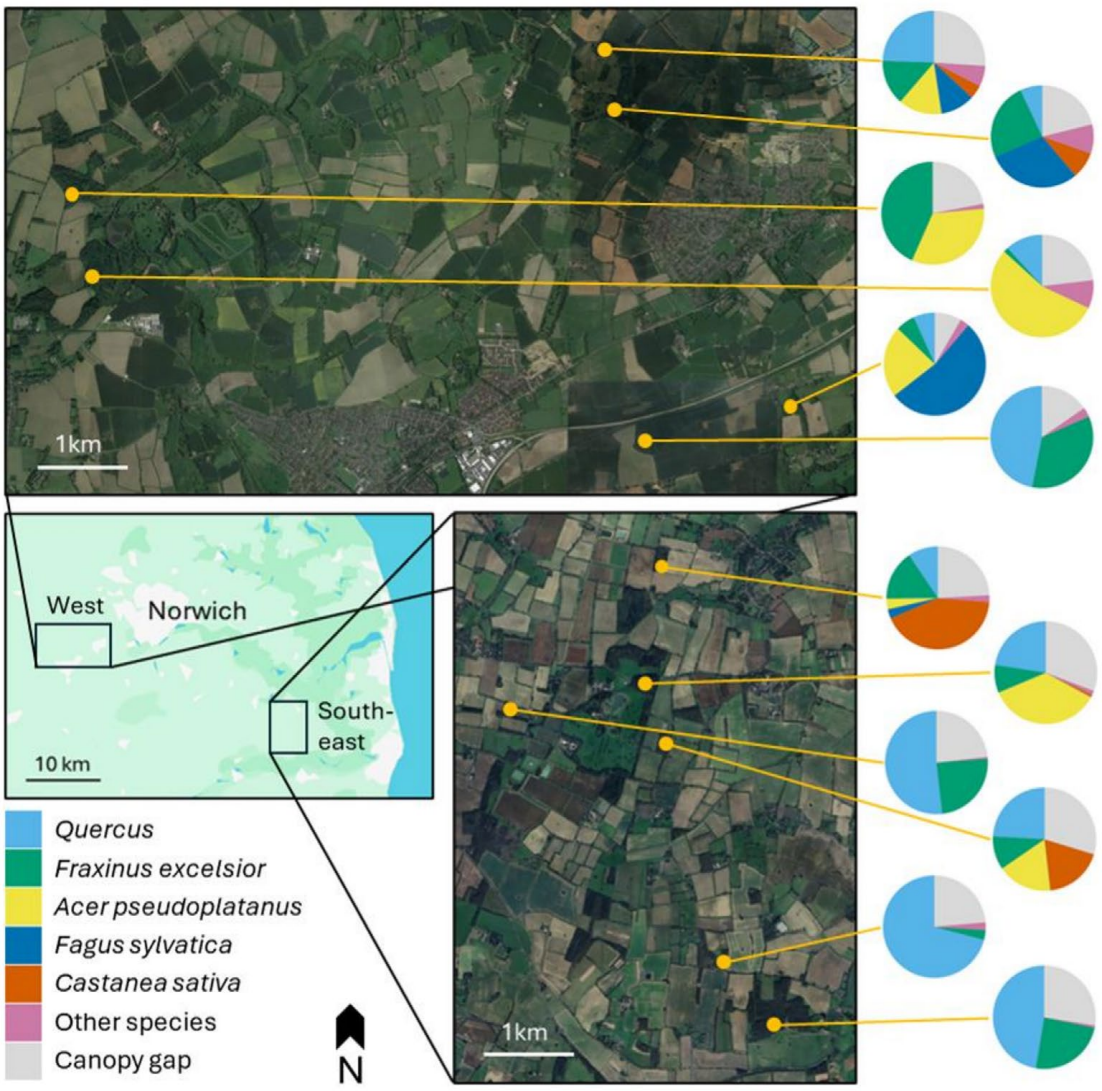
The two study regions in Norfolk, UK: ‘West’ and ‘South‐east’. The percentage areal extent of the crowns of each tree species within each woodland site (orange circles) is displayed (for further details, see Appendix S1). Site map as in Allen et al. (2025).

A map‐based search was conducted to identify privately owned Norfolk woodlands that are mature (majority of trees > 20 m in height: Lidar maps from Norfolk Trees and Hedges (Norfolk County Council 2022)), deciduous (identified from Natural England's Priority Habitat Inventory) and bordered by arable farmland. Twelve woodlands were selected that were representative of those available from the identified sites (Figure 1) and additionally met the criteria set by Allen et al. (2025) (interspersed presence/absence of *Acer pseudoplatanus* L.). Woodlands were all managed under the UK government's English Woodland Grant Scheme: most had undergone some tree thinning and contained tracks for vehicular access. All woodlands provided understory floral resources, though their abundance and type varied considerably across sites (Figure S1).

Sites clustered into two regions, ‘West’ and ‘South‐east’ Norfolk, separated by 23 km (Figure 1). Within each region, sites were separated from their nearest neighbour by ≥ 500 m (a compromise to ensure adequate site independence, commonly considered to be fully achieved at 1 km, under logistical considerations and limited site availability).

### 2.2 Bee Sampling Regime

BanfieldBio blue vane traps (BVTs) were used to catch bees. These can effectively sample the bee community, especially bumblebees, and their design is well‐suited to canopy sampling (Allen and Davies 2023). Traps were partially filled with water to euthanise and store specimens. Woodlands were sampled at three to five trapping locations, depending on their size, for an average of four locations per woodland. For unbiased selection of canopy trap locations, areas of mature (> 20 m height) deciduous woodland were searched for the first available tree with suitable crown‐branches for rigging, while constraining inter‐trap distance to between 60 and 90 m. A Bigshot slingshot with a weighted throwline was used to rig ropes to which traps were attached to be raised into the canopy. Traps were set towards the edge of tree crowns within the top third of the total tree height (i.e., at > 13.5 m in height). Understory traps were set in the same locations, each beneath its corresponding canopy trap. Adjacent to each woodland, two traps, separated by 60 m, were set at a nearby hedged field boundary, facing a non‐insect‐attractive crop or, in one case, pasture. In both habitats, traps were set at a height of ca. 0.8 m, with field margin traps at a distance of 0.5 m from the boundary‐hedge on its southern side.

BVTs were set over four sampling periods in 2022: the first, 5–11 May (‘early May’); the second, 26–30 May (‘late May’); the third, 15–18 June (‘mid‐June’); and the fourth, 11–16 July (‘mid‐July’). As such, we sampled in late spring (May) and summer (June/July), both before and after canopy closure (the point at which most trees have fully opened their leaves), which occurred towards the end of May. Weather forecasts were monitored to ensure sampling periods coincided with suitable weather for bees (mostly dry and sunny; minimum daily temperature high of 14°C; maximum wind speeds of 7.5 m s^−1^). For each sampling period, traps were set one woodland site at a time over two days (one day per region), and at the end of each deployment, traps were removed in the same order and over the same time frame. Thus, within each sampling period, all traps were deployed for approximately the same number of hours (ca. 120, 76, 48 and 96, respectively), and traps within each site were deployed over the same period of time (and, hence, weather conditions). Note that one canopy trap and one understory trap failed in mid‐June and mid‐July, respectively.

During each sampling period, ground floral cover was estimated across a 100 m^2^ quadrat centred on each ground‐level trap (see Appendix S1 for further details). Previously, this measure was found to have no relationship with ground‐level trap catches of all bees (Allen et al. 2025), and we confirmed its non‐significance in the present study by adding it, post hoc, to models of species and caste trap‐catch (see Appendix S3 for further details).

Weather records were taken from the nearest available location (Norwich Weather Centre) to the sampling sites (Past Weather, Norwich, May–July 2022). Average temperature was calculated from hourly records between 06:20 and 20:20 over the complete sampling days for each region in each period. All bee specimens were pinned and stored, with tags linking specimens to their samples. *Bombus* specimens were identified to species by the lead author using Falk (2015). Eye parameter measurements (μm.rad) for each species were taken from Tichit et al. 2024. Using Fiji (ImageJ) software, intertegular distances (mm) of worker specimens were measured from images taken with Zen 2.6 (blue edition) software under 6× (0.6 × 10) magnification. Workers of each species were measured if, within a given period, there were a minimum of 10 specimens in each of at least two habitats. We use intertegular distance as a proxy for body size as the former closely predicts the latter in worker bumblebees (Hagen and Dupont 2013).

### 2.3 Statistical Analyses

Analyses were conducted in R, version 4.3.2. Five sets of response variables of the social bumblebees were modelled (Table 1): (1) trap catch of individual species; (2) community weighted mean (CWM) of the eye parameter; (3) trap catch of reproductive and worker castes; (4) proportion of reproductives (weighted by sample size); and (5) intertegular distance of worker individuals. All were modelled as a function of *habitat* (2–3 levels: Field margin, Understory and/or Canopy) and initially fitted with *trap* (20–120 levels, nested within *site*), where necessary, and *site* (10–12 levels) as random intercepts to account for the non‐independence of specimens within the same trap, and traps/samples within the same site, respectively. For all response variables, each period was modelled separately to avoid excluding factor and grouping levels that were not present across all periods, to correct diagnostic issues where they presented, and for ease of model interpretation. Omnibus tests were performed on *habitat* and *habitat* interactions using likelihood ratio *χ* ^2^ (LRT) (models 1–8 and 13–21: Table 1) and ANOVA (9–12 and 22–31). To correct for multiple testing, the Benjamini–Hochberg (BH) method was applied to three families of omnibus test *p* values, which comprised related response variables: (1) species abundance (Q1‐3: models 1–8, plus a correlation test), (2) caste abundance (Q4: models 9–21) and (3) body size (Q5: models 22–31).

**TABLE 1:** Summary of all statistical models, grouped by investigated question (Question no.) and response‐variable set (Response no.).

Question no.	Model no.	Dataset					Sites (*n*)	Traps (*n*)	Response no.	Model equation (R notation)	Obs. (*n*)
		Sampling period	*Bombus* species	Field margin	Understory	Canopy					
1	1	Early May	6 most abundant	✓	✓	✓	12	120	1	Species catch ~ habitat*species + (1|site/trap)	720
	2	Late May	6 most abundant	✓	✓	✓	12	120		Species catch ~ habitat*species + (1|site/trap)	720
	3	Mid‐June	6 most abundant	✓	✓	✓	12	119		Species catch ~ habitat*species + (1|site/trap)	714
	4	Mid‐July	5 most abundant	✓	✓	✓	12	119		Species catch ~ habitat*species + (1|site/trap)	595
3	5	Early May	6 most abundant	✓	✓	✓	12	n/a	2	CWM of eye parameter ~ habitat + (1|site)	36
	6	Late May	6 most abundant	✓	✓	✓	12	n/a		CWM of eye parameter ~ habitat	36
	7	Mid‐June	6 most abundant	✓	✓	✓	12	n/a		CWM of eye parameter ~ habitat + (1|site)	33
	8	Mid‐July	6 most abundant	✓	✓	✓	12	n/a		CWM of eye parameter ~ habitat + (1|site)	36
4	9	Early May	All non‐parasitic	✓	✓	✓	12	120	3	Caste catch ~ habitat*caste + (1|site/trap)	240
	10	Late May	All non‐parasitic	✓	✓	✓	12	120		Caste catch ~ habitat*caste + (1|site/trap)	240
	11	Mid‐June	All non‐parasitic	✓	✓	✓	12	119		Caste catch ~ habitat*caste + (1|site/trap)	238
	12	Mid‐July	All non‐parasitic	✓	✓	✓	12	119		Caste catch ~ habitat*caste + (1|site/trap)	238
	13	Early May	*pascuorum*	✓	✓	✓	12	n/a	4	cbind(reproductives, workers) ~ habitat + site	33
	14	Early May	*pratorum*	✓	✓	✓	12	n/a		cbind(reproductives, workers) ~ habitat + (1|site)	30
	15	Late May	*pascuorum*	✓	✓	✓	10	n/a		cbind(reproductives, workers) ~ habitat	28
	16	Late May	*pratorum*	✓	✓	✓	12	n/a		cbind(reproductives, workers) ~ habitat + (1|site)	35
	17	Late May	*hypnorum*	✓	✓	✓	11	n/a		cbind(reproductives, workers) ~ habitat + site	25
	18	Late May	*hortorum*	✓	✓	✓	12	n/a		cbind(reproductives, workers) ~ habitat + site	28
	19	Mid‐June	*pratorum*	✓	✓		12	n/a		cbind(reproductives, workers) ~ habitat + (1|site)	22
	20	Mid‐July	*lapidarius*	✓		✓	12	n/a		cbind(reproductives, workers) ~ habitat + site	19
	21	Mid‐July	*terrestris*	✓	✓	✓	12	n/a		cbind(reproductives, workers) ~ habitat	31
5	22	Early May	*pratorum*	✓	✓	✓	12	60	5	Intertegular distance ~ habitat + (1|trap)	103
	23	Late May	*pratorum*	✓	✓	✓	12	76		Intertegular distance ~ habitat + (1|site/trap)	403
	24	Late May	*pascuorum*	✓	✓	✓	10	41		Intertegular distance ~ habitat + (1|site/trap)	78
	25	Late May	*lapidarius*	✓		✓	11	20		Intertegular distance ~ habitat	52
	26	Late May	*hypnorum*	✓	✓	✓	11	39		Intertegular distance ~ habitat + (1|trap)	59
	27	Late May	*hortorum*	✓	✓	✓	12	42		Intertegular distance ~ habitat + (1|trap)	87
	28	Mid‐June	*pratorum*	✓	✓		12	45		Intertegular distance ~ habitat + (1|site/trap)	101
	29	Mid‐July	*pascuorum*	✓	✓	✓	12	38		Intertegular distance ~ habitat + (1|site/trap)	51
	30	Mid‐July	*lapidarius*	✓		✓	11	26		Intertegular distance ~ habitat	66
	31	Mid‐July	*terrestris*	✓		✓	12	29		Intertegular distance ~ habitat + (1|trap)	49

Abbreviations: CWM = community weighted mean, n/a = not applicable.

Using the functions *glmmTMB* (package ‘glmmTMB’: Brooks et al. 2017) and *check_overdispersion* (package ‘performance’: Lüdecke et al. 2021), Poisson distributions in count data models (1–4 and 9–12: Table 1) were replaced with negative binomial distributions (family *nbinom2*) if there was significant overdispersion (model 2 only). For GLMs (1–4 and 9–21), and non‐parametric (5–8) and linear models (22–31), pairwise contrasts between levels of *habitat* were evaluated using *z*‐ and *t*‐tests, respectively (package ‘emmeans’: Lenth 2025). For models 1–4 and 9–12, contrasts were respectively limited to within species and within castes. For models including all three levels of *habitat*, *p* values were adjusted using Tukey's method. For linear and non‐parametric mixed‐effects models (5–8, 22–24, 26–29 and 31), degrees of freedom were estimated with the Kenward‐Roger method for both omnibus and contrast tests. All parametric models (1–4 and 9–31) were tested for diagnostic issues, including outliers and homogeneity of variance, and spatial autocorrelation, using ‘DHARMa’ (Hartig 2024) for GLMs, ‘performance’ for linear models, and ‘ncf’ (Bjornstad 2022) for all models. No model had significant diagnostic issues or problematic spatial dependence of its residuals (see Appendix S2 for further details).

### 2.4 Relative Activity of Species Across Habitats (Q1 and Q2)

Trap catches of bumblebees were split by species, and models 1–4 (Table 1) were run to examine relative activity levels in each habitat. Analyses were limited to species (including parasitic) represented by a minimum of 14 specimens in each period for three or more periods (Table 2, only social species shown). The strength of understory association for each species was calculated by subtracting field margin model estimates from understory estimates and averaging over all periods in which a species was modelled (Table S1). To visualise patterns of activity, species were ordered from the most understory associated to the least, and field margin and canopy estimates were plotted relative to understory estimates (which were set to zero). The relationship, within and across periods, between field margin and canopy estimates (both relative to understory) was tested using Pearson's correlation.

**TABLE 2:** Summary of social bumblebee species counts in each sampling period (with percentages in bold and in parentheses).

Social *Bombus* species	Sampling period				Total across sampling periods
	Early May	Late May	Mid‐June	Mid‐July	
*pratorum*	119 (**38**)	614 (**53**)	130 (**47**)	33 (**11**)	896
*pascuorum*	95 (**30**)	103 (**8.8**)	49 (**18**)	57 (**19**)	304
*hortorum*	23 (**7.3**)	199 (**17**)	34 (**12**)	38 (**12**)	294
*terrestris*	23 (**7.3**)	91 (**7.8**)	21 (**7.5**)	84 (**27**)	219
*lapidarius*	39 (**12**)	70 (**6.0**)	23 (**8.2**)	86 (**28**)	218
*hypnorum*	14 (**4.5**)	77 (**6.6**)	18 (**6.5**)	6 (**1.9**)	115
*lucorum*	0 (**0.0**)	8 (**0.7**)	4 (**1.4**)	4 (**1.3**)	16
*jonellus*	0 (**0.0**)	3 (**0.3**)	0 (**0.0**)	0 (**0.0**)	3
Total	313 (**100**)	1165 (**100**)	279 (**100**)	308 (**100**)	2065

### 2.5 Community Weighted Means of the Eye Parameter in Relation to Habitat (Q3)

The community weighted means (CWM) of bumblebee eye parameters were calculated, separately for each period, from the pooled trap catches of the six abundant species (from Q1 and Q2) from each habitat at each site. Models 5–8 (Table 1) were run to investigate the relationship between the CWM of the eye parameter and habitat. Linear mixed‐effects models were initially fitted but suffered from unequal variance and outliers, both when periods were modelled simultaneously and separately. We therefore used the rank‐based non‐parametric test Aligned Rank Transform (function *art*; package ‘ARTool’: Kay et al. 2025), which can accommodate random effects. *Site* was removed as a random effect if it caused problems with model convergence (model 6).

### 2.6 Relative Activity of Castes Across Habitats (Q4)

Trap catches were split by caste—between reproductives (queens and/or males) and workers—across all non‐parasitic bumblebee species, and models 9–12 (Table 1) were run to examine activity levels in each habitat. For species‐level analyses of caste ratios using logistic regression (models 13–21), trap catches were first pooled within each habitat at each site. To avoid issues of model convergence, analyses were limited to species with sufficient counts in a given period (when two or more habitats had a minimum count of 15 in each; Table S7, and when each caste grouping numbered no fewer than 15 across these habitats), resulting in nine suitable analyses (models 13–21; Table S6). *Site* was converted to a fixed factor if its initial fitting resulted in problems with model convergence (models 17, 18 and 20); it was then removed if it had little impact on model estimates (conservatively, if *p* values from *site* LRTs were > 0.15: models 15 and 21, with *p* = 0.64 and *p* = 0.73, respectively).

### 2.7 Worker Body Size in Relation to Habitat (Q5)

Models 22–31 (Table 1) were run to examine the species‐level relationship between worker body size and habitat, with modelling limited to habitats in which a minimum of 10 specimens were available. Within periods, species were modelled separately to avoid violations of homogeneity of variance. *Site* and *trap* were removed consecutively if their inclusion caused problems with model convergence. Where significant contrasts were found, any detected outliers (function *check_outliers*; package ‘performance’) were temporarily removed to ensure that they did not change the identification of significant contrasts (models 22 and 31).

## 3 Results

A total of 2065 social bumblebees were caught across the four sampling periods. The numbers of each species caught are summarised in Table 2.

### 3.1 Relative Activity of Species Across Habitats (Q1 & Q2)

**FIGURE 2:**
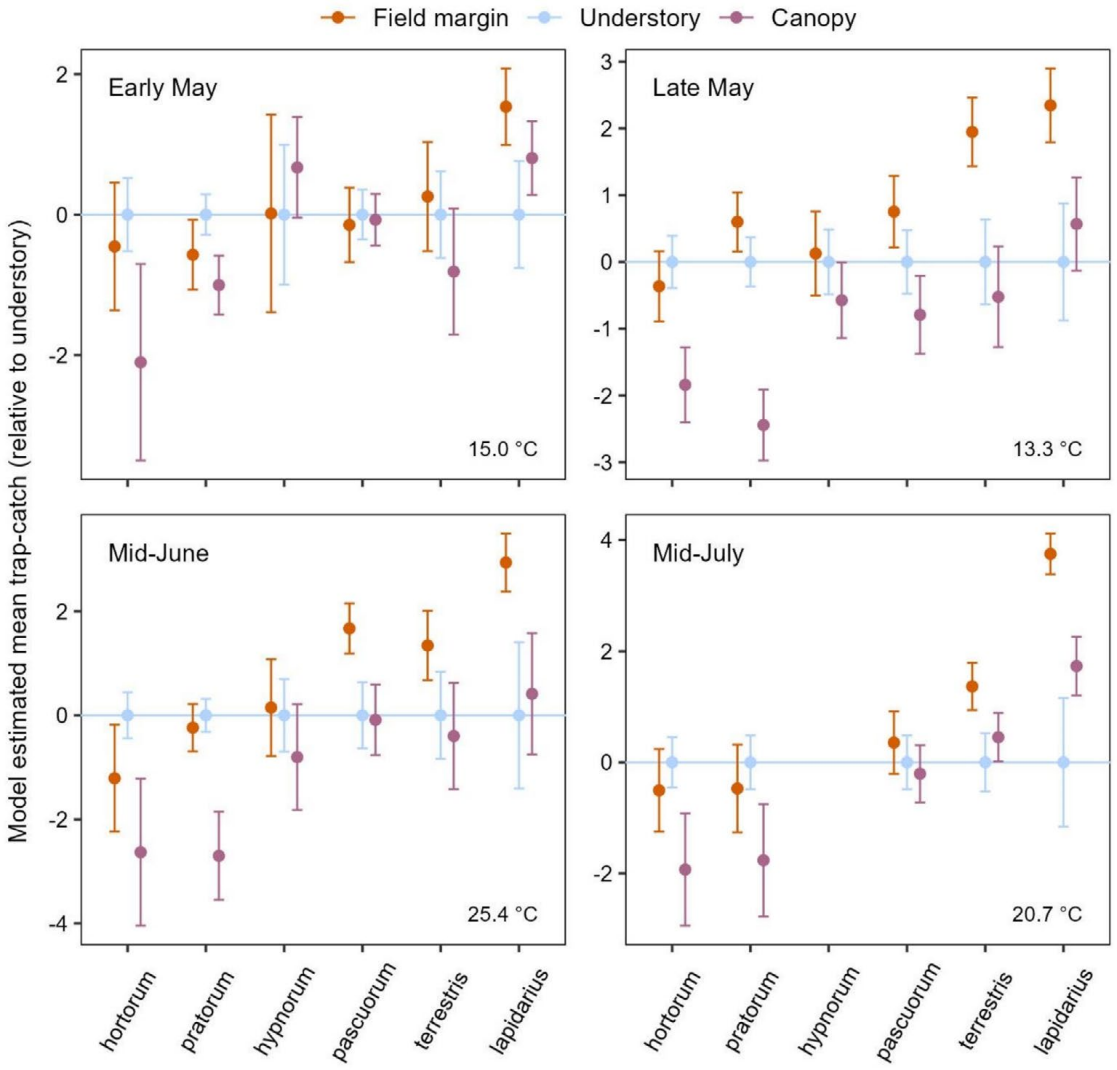
Relative model‐estimated means of *Bombus* species‐abundance per trap (log scale) in field margin, understory and canopy habitats over four sampling periods. Model estimated means are plotted relative to the understory to aid comparisons of relative activity patterns between species and sampling periods. Species are ordered along the *x*‐axis from the most understory‐associated to the least (relative to the field margin, and averaged across periods, Table S1). Error bars represent 95% confidence intervals. See Table S2 for pairwise contrasts of habitat within each species. Average temperature over full sampling days for each period is displayed.

Relative activity across the habitats was highly species‐specific: *habitat* × *species* interactions were highly significant (*p* < 0.001) in each sampling period (Table S2). Species' patterns of understory association (understory activity relative to field margin activity) were broadly consistent across the sampling periods (Figures 2 and S3), with *B. hortorum* and *B. lapidarius* having the highest and lowest understory associations (averaged across sampling periods, Table S1), respectively. In each period, the model estimated mean trap‐catch of *B. hortorum* was higher in the understory than at field margins, while the reverse was true for *B. lapidarius* , whose trap catches were significantly higher at field margins (Table S2). Otherwise, from most to least understory‐associated, the species are ordered as follows: *B. pratorum* , *B. hypnorum* , *B. pascuorum* and *B. terrestris* .

Species with higher field margin activity also tended to have higher canopy activity: relative to understory estimates, field margin and canopy estimates were positively correlated within each period, with the strength of correlation increasing with each successive period (*r*_4_ = 0.67, *r*_4_ = 0.70, *r*_4_ = 0.91 and *r*_3_ = 0.95, respectively). Across all periods, there was a strong and significant positive correlation between these estimates (Figure S3; *r*_21_ = 0.75, *p* < 0.001, with BH correction). Finally, during the hottest period in mid‐June (Figure 2; daily maximums of 27°C and 32°C, respectively, which meet/exceed the regional heatwave threshold [Met Office 2025]), the understory association of *B. hortorum* more than doubled (×2.16) relative to the other periods, while those of the other species decreased or increased only minimally ( *B. pratorum* : ×1.09).

### 3.2 Community Weighted Means of the Eye Parameter in Relation to Habitat (Q3)

**FIGURE 3:**
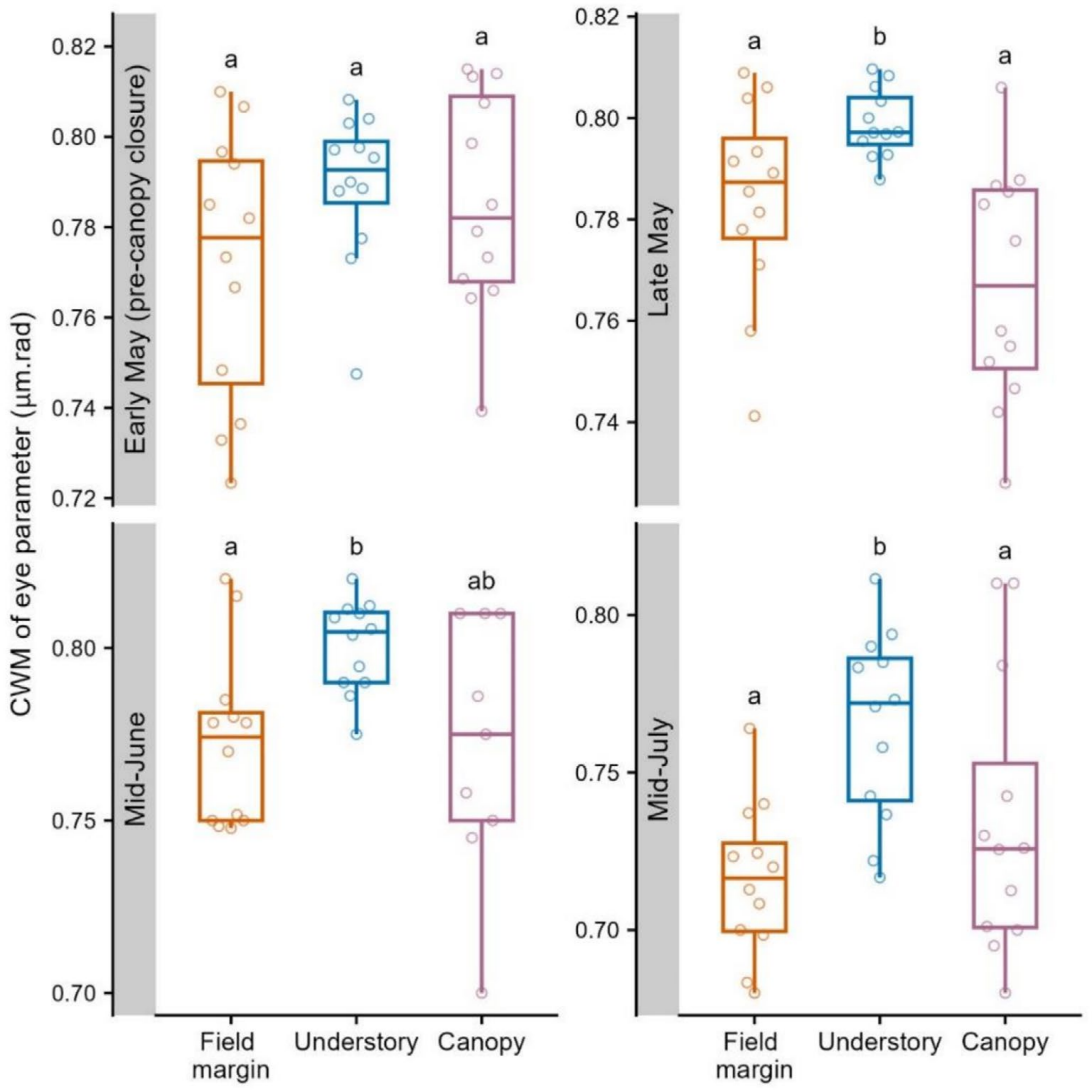
Community weighted means (CWM) of bumblebee eye parameter against habitat in each sampling period, based on the six most abundant species (Figure 2). Different letters denote significant (*p* < 0.05) differences between habitats within each period (see Table S3 for pairwise contrasts).

In early May, before canopy closure, CWM of the eye parameter did not differ significantly between the habitats (Figure 3 and Table S3). However, after canopy closure, from late May, CWM of the eye parameter in the understory was significantly higher than that of field margins in all sampling periods, and of canopies in late May and mid‐July.

### 3.3 Relative Activity of Castes Across Habitats (Q4)

**FIGURE 4:**
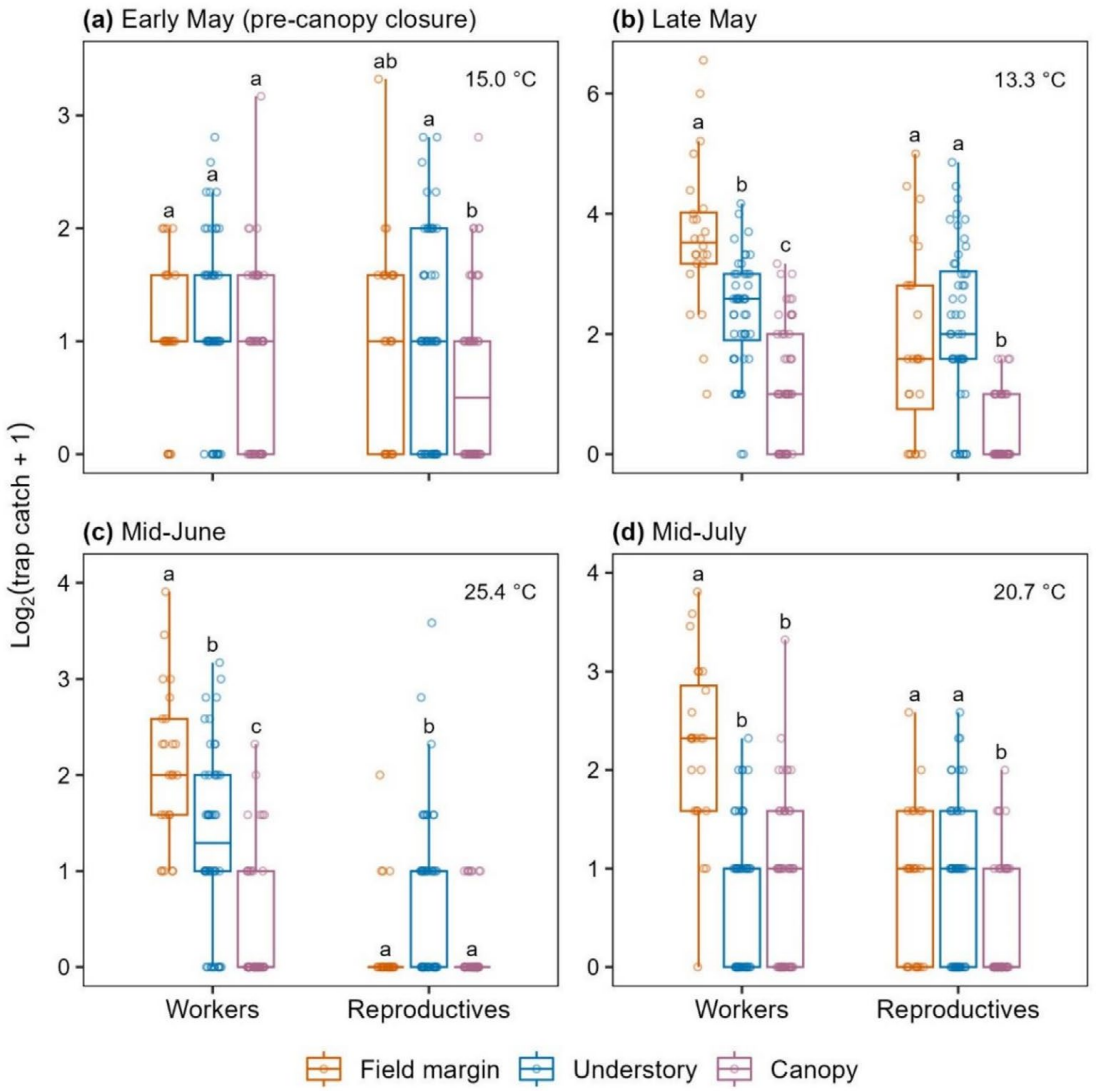
Trap catches of bumblebee workers and reproductives (queens and/or males) from each habitat in each sampling period. Different letters denote significant (*p* < 0.05) differences between habitats within each caste grouping in each period (see Table S4 for pairwise contrasts). Average temperature over full sampling days for each period is displayed.

In each sampling period after canopy closure (from late May), relative activity across the habitats was highly caste‐specific: *habitat* × *caste* interactions were highly significant (*p* < 0.001: Table S4). However, before canopy closure, in early May, this interaction was non‐significant. From late May, worker trap‐catches at field margins were significantly higher than those of understories in all sampling periods, while for reproductives, they were equal between field margins and understories (Figure 4b,d), except during the hottest period when they were significantly lower at field margins (Figure 4c).

**FIGURE 5:**
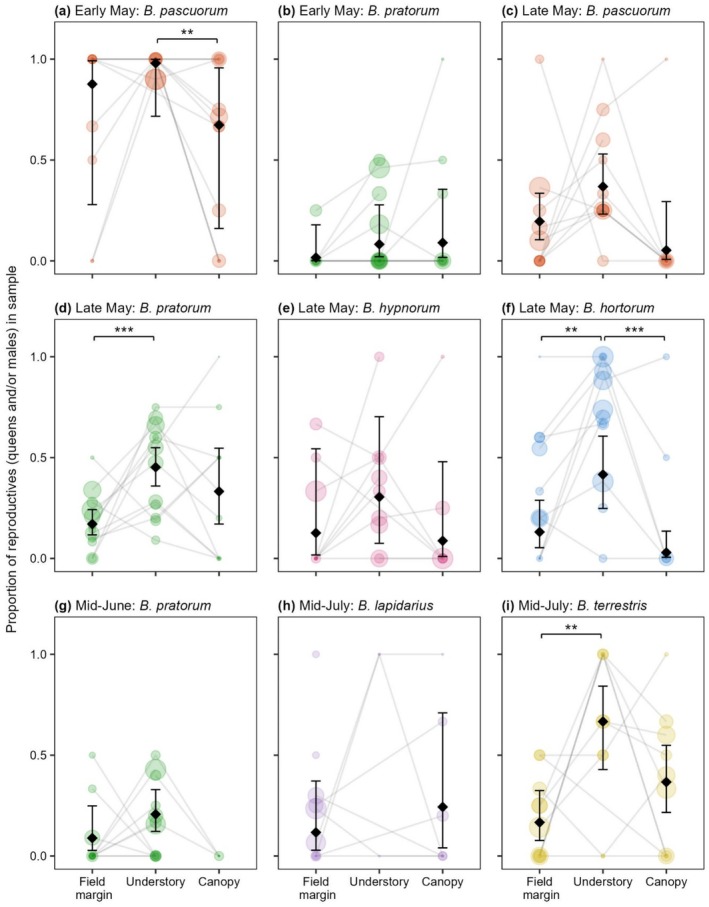
The proportion of reproductives in each habitat for all period–species pairings in which sufficient specimens were caught for caste‐ratio analysis (see Methods). The size of coloured circles represents the relative sample size within each period–species pairing. Samples are the pooled catches of field margin, understory and canopy traps, respectively, within each site (see Table S6 for caste counts and sample sizes). Grey lines connect samples within the same site. Model estimates are superimposed (black diamonds) with error bars representing 95% confidence intervals. Significant differences between caste ratios are indicated as follows: ***p < 0.001 and **p < 0.01 (see Table S5 for all pairwise contrasts).

For *B. pratorum* and *B. hortorum* in late May, and *B. terrestris* in mid‐July, caste ratios were significantly skewed towards reproductives in the understory relative to field margins (Figure 5d,f,i and Table S5). Relative to the canopy, this skew was also significant for *B. pascuorum* in early May and *B. hortorum* in late May (Figure 5a,f). Considering total counts of the six abundant bumblebee species in each period, the percentage of reproductives in the understory is higher than that of field margins in 22 out of 23 instances, and higher than that of canopies in 19 out of 24 instances (Table S7).

### 3.4 Worker Body Size in Relation to Habitat (Q5)

**FIGURE 6:**
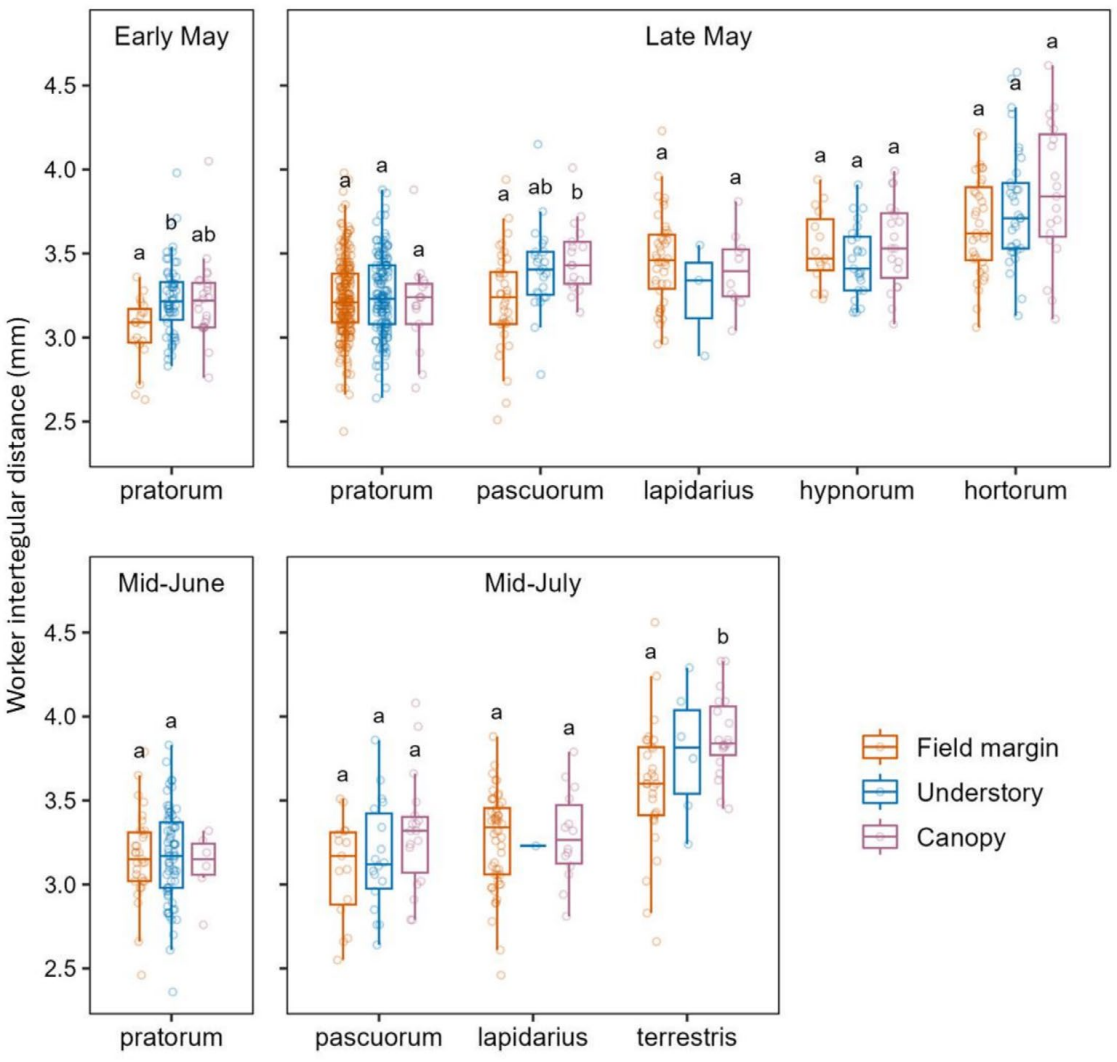
Intertegular distance of *Bombus* workers according to species, habitat and sampling period. Analyses are limited to period–species pairings in which sufficient specimens were caught (> 9 in each habitat; see Methods). Different letters denote significant (*p* < 0.05) differences between habitats within each species in each period (see Table S8 for pairwise contrasts).

We found weak evidence for a relationship between habitat and worker body size: after BH correction, habitat effects were near‐significant (*p* < 0.1) for three species (Table S8). Post hoc contrasts indicated that, compared to field margins, workers were larger in the understory for *B. pratorum* in early May, and in the canopy for *B. pascuorum* in late May and *B. terrestris* in mid‐July (Figure 6). For the remaining species in late May, mid‐June and mid‐July, there were no significant differences in worker size between the habitats.

## 4 Discussion

From late spring to mid‐summer, we found broadly consistent patterns of woodland understory association (understory activity relative to that of field margins) among the common bumblebee species, with *B. hortorum* and *B. lapidarius* being the most and least understory‐associated, respectively (Figure 2). An affinity for the understory is likely influenced by a species' visual performances under low light conditions: relative activity levels across species in the two sun‐exposed habitats were highly positively correlated, and understory bumblebee communities had higher average light sensitivity (Figure 3). After canopy closure, the reproductive castes were more understory‐associated than workers (Figure 4), and *B. pratorum* , *B. hortorum* and *B. terrestris* were identified as contributing to this pattern (Figure 5). Additionally, larger worker bees may have been more active in the woodland habitats, but this did not apply to all species and was inconsistent across sampling periods (Figure 6). Finally, when temperatures met heatwave thresholds (Met Office 2025) in mid‐June, understory associations at the species‐ and caste‐level changed: that of *B. hortorum* doubled, and that of reproductives, but not workers, increased across all species.

We sampled bees with BVTs, which may attract individuals from distances up to tens of metres. Consequently, trap catches are likely, on average, to be biased downwards in woodland habitats, relative to field margins, by nearby vegetational structures obscuring traps from view (Allen et al. 2025). Therefore, average trap catches between the habitats sampled in the present study cannot provide absolute measures of bumblebee activity‐density in each habitat for comparison. Rather, differences in patterns of relative abundance‐per‐trap between species, castes and different sampling periods are informative.

### 4.1 Species Habitat Associations

We found that relative levels of activity across the habitats were highly species‐specific. Overall, *B. hortorum* and *B. pratorum* were the most understory‐associated species, while *B. lapidarius* and *B. terrestris* were the least and *B. pascuorum* and *B. hypnorum* were intermediate (Figure 2). The results of previous studies are largely consistent with our own. When included, *B. lapidarius* is the least associated with woodlands/woodland interiors, followed by *B. terrestris* (Allen and Davies 2023; Whitehorn et al. 2022; Tichit et al. 2024). However, *Bombus pascuorum* frequently shows positive associations with woodland, which may be attributed to the woodland‐edge habitat (Svensson et al. 2000; Sõber et al. 2020; Maurer et al. 2022; Tichit et al. 2024; but see Whitehorn et al. 2022). Allen and Davies (2023) found *B. hortorum* and *B. pratorum* to have the highest relative abundance in woodland interiors in late spring, while Tichit et al. (2024) found that *B. pratorum* abundance in grasslands increased with surrounding woodland cover. Our study highlights the importance of woodlands to these latter two species. Indeed, given that understory traps likely underrepresent bumblebee density in this habitat, woodland activity of these species could be considerably higher than that of field margins for much of the late spring to mid‐summer period.

A previous analysis of total wild bee abundance found that after canopy closure and relative to the understory, activity levels in the two more sun‐exposed habitats—canopy and field margin—were highly positively correlated, with the suggestion that canopy activity was largely a function of bees avoiding the understory while traversing woodlands (Allen et al. 2025). Here, when examining the common social bumblebees separately, we find that this correlation is confirmed and generalised to include the period before canopy closure, with the strength of correlation increasing with each successive sampling period. This suggests that species differ in their response to shade, and therefore their likelihood of entering the understory, as light levels decrease with the progressing season.

We found that the CWM of the eye parameter was higher in the understory relative to the other habitats after canopy closure (Figure 3), that is, there were relatively more individuals of light‐sensitive species in the shaded understory than in the other, sun‐exposed habitats. Similarly, Bartholomée et al. (2023) found that light‐sensitive species were more active under darker conditions in hemi‐boreal forest, while Tichit et al. (2024) found the CWM of the eye parameter of grassland bumblebee communities in Sweden increased with surrounding forest cover. The eye parameter appears to be consistent within species; however, measures are based on just a few worker specimens (Tichit et al. 2024), and confidence in our interpretations would be improved if sample sizes were bigger and included males and queens. Finally, we note in the present study that the ordering of species' understory associations does not match their respective eye parameters (Table S1). Importantly, however, light sensitivity also increases with body size (Fitzgerald et al. 2022), such that *B. hortorum* and *B. terrestris* , whose workers are larger on average, may be more light‐sensitive than *B. pratorum* / *B. pascuorum* and *B. lapidarius* , respectively, despite having slightly lower eye parameters than each respective group.

Nonetheless, other factors probably influence affinities for the understory. Tubular flowers were more abundant in woodlands than at field margins (Figure S1), and *B. hortorum* —the species with by far the longest tongue (Goulson et al. 2008)—would be best adapted to exploit these resources. However, the next most understory‐associated species, *B. pratorum* , also has the shortest tongue. Larger bumblebees may be better able to maintain optimal body temperatures under cooler conditions (Feuerborn et al. 2023), such as those of woodland understories. However, while *B. hortorum* workers are among the largest, those of *B. pratorum* and *B. pascuorum* are among the smallest (Peat, Darvill, et al. 2005). Species with low heat tolerance may be more forest‐associated (Feuerborn et al. 2023). Indeed, *B. hortorum* has the lowest heat tolerance in the present study; however, that of *B. pratorum* is high, while that of *B. lapidarius* is relatively low (Martinet et al. 2021). Finally, species differ in certain aspects of their ecology—for example, in their floral (Goulson et al. 2008) and nesting (Kells and Goulson 2003; Pugesek et al. 2024) preferences, and in the micro‐site features visited by scent‐marking males (Bringer 1973; Fussell and Corbet 1992)—which may not relate to any morphological or physiological trait, but could nonetheless strongly influence their affinity for woodlands.

### 4.2 Caste Habitat Associations

We also found that relative levels of activity across the habitats were highly caste‐specific after canopy closure. From late May, the reproductive castes were significantly more understory‐associated than workers (Figure 4). Similarly, Whitehorn et al. (2022) found that *B. pratorum* and *B. lucorum* reproductives, but not workers, had significant, positive associations with woodland. We detected significant skews towards reproductives in the understory for *B. pascuorum* , *B. pratorum* , *B. hortorum* and *B. terrestris* (Figure 5). Additionally, considering total counts of each of the bumblebee species in each period, the percentage of reproductives in the understory was almost always higher than that of field margins and canopies (Table S7), indicating that understory skews towards reproductives could be a general feature of bumblebee activity patterns.

Except for *B. pascuorum* , significant skews were detected when the populations of reproductives were largely represented by males (Table S6). Males may differ from females in their floral preferences (Roswell et al. 2019), which could influence habitat selection; however, we consider a more plausible explanation for caste skews comes from the distinct patrolling behaviour of males as they seek unmated queens for copulation. While all castes will tend to find more floral resources at field margins (Figure S2), woodlands may provide the micro‐site features required for scent‐marking and patrolling males. For example, *B. hortorum* scent marks the bases of tree trunks and *B. pratorum* marks fronds of vegetation from ground level up to a height of 4 m (Bringer 1973), and skews towards males in the understory relative to the canopy have previously been detected for these species (Allen and Davies 2023). Furthermore, in the present study, understory trap catches of reproductives were equal to or greater than those of field margins from late May. Given the potential to underestimate understory bee density and the greater availability of forage at field margins, this suggests that patrolling activity could be considerably higher in woodland understories than at field margins.

In early May, however, males were scarce (Table S9), and all *B. pascuorum* reproductives were represented by queens (Table S6). In spring, many queens are seeking microhabitats in which to start their colonies, and woodland understories can provide an abundance of these (O'Connor et al. 2017; Mola et al. 2021; Pugesek and Crone 2022; Pugesek et al. 2024). Queens may also be better suited for foraging in the understory than workers. They have larger bodies that retain heat more easily, larger eyes that provide greater light sensitivity and longer tongues (Fitzgerald et al. 2022) that may be more efficient at handling the abundant tubular flowers of understories.

### 4.3 Worker Body Size in Relation to Habitat

We provide equivocal evidence that, relative to field margins, the workers of some species were larger in the understory during particular sampling events (Figure 6). To our knowledge, no other studies have investigated habitat segregation of bumblebee workers according to body size. Larger individuals may travel greater distances (Ohashi et al. 2008) and can forage in dimmer conditions (Hall et al. 2021) and on flowers with deeper corollas, while smaller ones forage more efficiently on shallower flowers (Peat, Tucker, and Goulson 2005). However, there is no clear evidence that worker body size influences thermal tolerance (reviewed in Fitzgerald et al. 2022). Greater light sensitivity, longer tongues and potentially greater cold tolerance could, together or separately, lead to larger average worker size in woodland understories. Additionally, larger bees may forage over greater distances and therefore be more likely to traverse woodlands, via the canopy, in search of flowers in open habitats. Which mechanisms are at play likely depends on the time of year and the species in question and could also be influenced by floral availability and weather conditions, leading to the inconsistent appearance of larger workers in woodland habitats across the analysed species.

### 4.4 Possible Temperature Effects

Across sampling events, field margin floral abundance remained constant (Figure S2), and this was likely the case for adjacent field interiors also, as none contained insect‐attractive crops. However, there was significant temperature variation, and during the mid‐June sampling event, temperatures met heatwave thresholds (Met Office 2025), which appears to have impacted bumblebee activity. Relative to the field margins after canopy closure, understory activity significantly increased for reproductives (mainly males: Table S9), but not for workers (Figure 4), and, in contrast to the other species, the understory association of *B. hortorum* more than doubled relative to the other periods (Figure 2). During the warmest weather, exposed insects—especially heat‐sensitive groups—must seek cooler microhabitats to avoid overheating (Sunday et al. 2014; Hayes et al. 2024). *Bombus hortorum* is considerably less heat‐tolerant than the remaining species (Martinet et al. 2021). Queens can be less heat‐tolerant than workers (but not always: Maebe et al. 2021), while there is no clear evidence of this in males (Feuerborn et al. 2023). However, males may reduce their activity at elevated temperatures that are nonetheless below the threshold for heat stupor: first, sperm viability declines before thresholds of organismal tolerance are reached (Campion et al. 2023); and second, male activity levels might correlate with those of the more heat‐sensitive unmated queens, which they spend considerable time attempting to attract during their patrols.

## 5 Conclusions, Conservation Implications and Future Directions

In this study, we show that the relative levels of activity in woodland interiors are highly caste‐ and species‐specific and that they also may vary according to worker size. In particular, we show that understory associations are highest for species with greater light sensitivity, for the reproductive castes, and possibly for larger workers, and that some of these associations may increase during hot weather. These patterns have been revealed by a novel survey design (Allen et al. 2025)—sampling both shaded and sun‐exposed woodland and open habitats across the season—which could be applied to other mobile heterotherms/ectotherms and pollinating insects in agricultural landscapes, providing insights into their woodland interactions, and how they co‐exist and persist under a warming climate.

The consistency of species' relative woodland associations across studies suggests this habitat has played a key role in directing niche differentiation, resulting in differential exploitation of woodlands among species—and support for their co‐existence—in present‐day agricultural landscapes. We bolster the correlational evidence that woodland and shade associations are driven, in part, by species' light sensitivity. Additionally, our findings suggest an important role for woodlands in facilitating reproductive behaviours by providing nesting and patrolling sites for nest‐seeking queens and queen‐seeking males, respectively. Finally, an affinity for woodlands may be greater for larger workers under some circumstances, potentially reducing intraspecific competition and facilitating the exploitation of the spatially and temporally variable resources surrounding colonies.

Woodlands, and potentially other forms of agroforestry, could be key to sustaining bumblebee abundance and diversity in farmed landscapes—particularly in the United Kingdom, one of the least wooded countries in Europe (Forest Research 2017). Specifically, woodlands may increase the relative abundance of bumblebees that peak early in the season (e.g., *B. pratorum* ) and have longer tongues (e.g., *B. hortorum* and *B. pascuorum* ), which could benefit early flowering crops (e.g., oilseed rape and fruiting trees) and leguminous crops (e.g., *Vicia faba* and *Trifolium pratense* : Burns and Stanley (2023)), respectively. Additionally, woodlands could have a protective role during heatwaves, providing refuge to bumblebees. However, it remains uncertain if, at high temperatures, bumblebees select more shaded environments for their usual activities, or alternatively, if more open habitat‐associated species become less active overall. The former scenario would indicate that, under a warming climate, management to increase tree cover would benefit the entire bumblebee community, while the latter would indicate increased resilience only of the more woodland‐associated species.

To investigate this question, it will be necessary to elucidate the impact of temperature versus light and shade on the community of foraging bumblebees, independent of habitat, which could be achieved with experimental manipulations in the field. To understand how woodlands may support the wider bee community, differences in the non‐corbiculate (solitary) bee assemblages between canopy, understory and open habitats should be examined. Finally, observational methods could be used to establish the relative frequency of nesting, foraging and patrolling behaviours, in order to better understand how woodland understories and field margins co‐function to support bumblebee communities and to maximise their respective benefits.

## Author Contributions

**Guthrie Allen:** conceptualization (equal), data curation (lead), formal analysis (lead), investigation (lead), methodology (equal), visualization (lead), writing – original draft (lead), writing – review and editing (lead). **Lynn V. Dicks:** methodology (supporting), supervision (supporting), writing – review and editing (supporting). **Martin I. Taylor:** methodology (supporting), supervision (supporting), writing – review and editing (supporting). **Daniel Hewitt:** supervision (supporting), writing – review and editing (supporting). **Richard G. Davies:** conceptualization (equal), funding acquisition (lead), methodology (equal), project administration (lead), supervision (lead), writing – review and editing (supporting).

## Funding

This work was supported by the Natural Environment Research Council, NE/S007334/1 Woodland Trust.

## Conflicts of Interest

The authors declare no conflicts of interest.

## Supporting information


**Appendix S1:** Tree and ground floral surveys.
**Appendix S2:** Model diagnostics.
**Figure S1:** Floral abundance of the six most common plant families at each ground‐level trap—see Appendix S1 for an explanation of floral index scores. Periods I, II, III and IV represent the sampling periods in early May, late May, mid‐June and mid‐July, respectively. Note that ‘Liliaceae’ comprises *Hyacinthoides non‐scripta* and *Allium ursinum* , which are now classified within Asparagaceae and Amaryllidaceae, respectively.
**Figure S2:** Combined floral abundance of all bee‐visited plant families at each ground‐level trap—see Appendix S1 for an explanation of floral index scores. Periods I, II, III and IV represent the sampling periods in early May, late May, mid‐June and mid‐July, respectively.
**Table S1:** Species' strength of understory association (mean of understory estimate minus field margin estimate across modelled periods; Table S2) and eye parameter (taken from Tichit et al. (2024)).
**Table S2:** Omnibus tests (likelihood ratio *χ* ^2^) and pairwise contrasts of species trap‐catch between habitats (models 1–4: Table 1). Omnibus test *p* values are adjusted using the Benjamini–Hochberg method for a family of nine tests (models 1–8, plus the correlation test). ‘Open’ = Field margin. Estimates are given on the log (not the response) scale. Contrast test *p* values are adjusted using the Tukey method for comparing a family of three estimates. *p* values < 0.05 are in bold.
**Figure S3:** The relationship between field margin and canopy model‐estimated mean bee abundance per trap (log scale), relative to the understory, across *Bombus* species and sampling periods (models 1–4).
**Table S3:** Omnibus tests (ANOVA) and pairwise contrasts of the ranked community‐weighted means of eye parameter between habitats (models 5–8: Table 1). Omnibus test *p* values are adjusted using the Benjamini–Hochberg method for a family of nine tests (models 1–8, plus the correlation test). ‘Open’ = Field margin. For mixed effects models (5, 7 and 8), degrees of freedom are estimated using the Kenward–Roger method. Contrast test *p* values are adjusted using the Tukey method for comparing a family of three estimates. *p* values < 0.05 are in bold.
**Table S4:** Omnibus tests (likelihood ratio *χ* ^2^) and pairwise contrasts of caste trap‐catch between habitats (models 9–12: Table 1). Omnibus test *p* values are adjusted using the Benjamini–Hochberg method for a family of 13 tests (models 9–21). ‘Open’ = Field margin. Estimates are given on the log (not the response) scale. Contrast test *p* values are adjusted using the Tukey method for comparing a family of three estimates. *p* values < 0.05 are in bold.
**Table S5:** Omnibus tests (likelihood ratio *χ* ^2^) and pairwise contrasts of the log odds ratio of reproductive (male and/or queen) presence between habitats (models 13–21: Table 1). Omnibus test *p* values are adjusted using the Benjamini–Hochberg method for a family of 13 tests (models 9–21). ‘Open’ = Field margin. Estimates are averaged over levels of *site* where this is included as a fixed factor (models 17, 18, 20). Where three habitats are included, contrast test *p* values are adjusted using the Tukey method. *p* values < 0.05 are in bold.
**Table S6:** Summary of bumblebee counts comprising the analyses presented in Figure 5. Reproductive and worker castes are in bold. F = Field margin; U = understory; C = Canopy.
**Table S7:** Total bumblebees sampled in each habitat according to species and period, with the percentage comprising reproductives (queens and/or males) in parentheses. Understory percentages are in bold and/or *italics* if they are larger than the field margin and canopy percentages, respectively. Only the six most abundant social bumblebee species are displayed.
**Table S8:** Omnibus tests (ANOVA) and pairwise contrasts of worker intertegular‐distance between habitats (models 22–31: Table 1). Omnibus test *p* values are adjusted using the Benjamini–Hochberg method for a family of ten tests. ‘Field’ = Field margin. For mixed effects models (22–24, 26–29, 31), degrees of freedom are estimated using the Kenward–Roger method. Where three habitats are included, contrast test *p* values are adjusted using the Tukey method. *p* values < 0.05 are in bold.
**Table S9:** Summary of social bumblebee caste counts in each sampling period (with percentages in parentheses).
**Appendix S3:** Post hoc testing of *floral index*.

## Data Availability

The data that support the findings of this study are openly available in Dryad at https://doi.org/10.5061/dryad.jwstqjqqb.
