# Supplementary material for: Woodlands Facilitate Reproductive Behaviour and Niche Partitioning in Farmland Bumblebee Communities
Source: Ecol Evol. 2026 Apr 13;16(4):e73415. doi: 10.1002/ece3.73415 (PMC13071471; doi:10.1002/ece3.73415)
Supplement: Supplementary file 1 — Appendix S1: Tree and ground floral surveys. Appendix S2: Model diagnostics. Figure S1: Floral abundance of the six most common plant families at each ground‐level trap—see Appendix S1 for an explanation of floral index scores. Periods I, II, III and IV represent the sampling periods in early May, late May, mid‐June and mid‐July, respectively. Note that ‘Liliaceae’ comprises Hyacinthoides non‐scripta and Allium ursinum , which are now classified within Asparagaceae and Amaryllidaceae, respectively. Figure S2: Combined floral abundance of all bee‐visited plant families at each ground‐level trap—see Appendix S1 for an explanation of floral index scores. Periods I, II, III and IV represent the sampling periods in early May, late May, mid‐June and mid‐July, respectively. Table S1: Species' strength of understory association (mean of understory estimate minus field margin estimate across modelled periods; Table S2) and eye parameter (taken from Tichit et al. (2024)). Table S2: Omnibus tests (likelihood ratio χ 2) and pairwise contrasts of species trap‐catch between habitats (models 1–4: Table 1). Omnibus test p values are adjusted using the Benjamini–Hochberg method for a family of nine tests (models 1–8, plus the correlation test). ‘Open’ = Field margin. Estimates are given on the log (not the response) scale. Contrast test p values are adjusted using the Tukey method for comparing a family of three estimates. p values < 0.05 are in bold. Figure S3: The relationship between field margin and canopy model‐estimated mean bee abundance per trap (log scale), relative to the understory, across Bombus species and sampling periods (models 1–4). Table S3: Omnibus tests (ANOVA) and pairwise contrasts of the ranked community‐weighted means of eye parameter between habitats (models 5–8: Table 1). Omnibus test p values are adjusted using the Benjamini–Hochberg method for a family of nine tests (models 1–8, plus the correlation test). ‘Open’ = Field margin. For mixed effects models ( [file ECE3-16-e73415-s001.docx]

**Supporting information for “Woodlands facilitate reproductive behaviour and niche partitioning in farmland bumblebees” (Appendices S1–S3; Figures S1–S3; Tables S1–S9).**

**Appendix S1**. *Tree and* *ground floral surveys*

Canopy tree species composition of each woodland (Figure 1) was determined by visually estimating the canopy-level areal extent of the crowns of each tree species at each 10 x 10 m section making up 20 x 20 m quadrats centred on each trap location and 20 x 40 m transects connecting adjacent trap locations (i.e., two, three and four transects in woodlands with three, four and five trap locations, respectively).

During each sampling period (extending a maximum of one day prior to trapping and three days post trapping), ground floral cover was estimated across a 100 m^2^ quadrat centred on each ground-level trap. Woodland understory quadrats were 10 x 10 m and field margin quadrats were 5 x 20 m. Flower cover – *not* vegetative cover – of each bee-visited plant family (DoPI, 2022) (with the exception of *Galium aparine* because of its minute flowers) was scored within each of the four 5 x 5 m sub-quadrats contained within each trap-quadrat using an adjusted Braun-Blanquet scale (scored 0.5, 1, 2, 3, 4 or 5, representing percentage covers of <1, 1 – 5, 5 – 25, 25 – 50, 50 – 75, 75 – 100, respectively). Scores summed over the sub-quadrats represented the floral score for the trap-quadrat (variable: *floral index*). The level of family was chosen because it is the taxonomic level most relevant to common polylectic bees in terms of dietary specialization (Wood et al., 2021).

**Appendix S2**. *Model diagnostics*

All generalised linear models (1–4 and 9–21: Table 1) were tested for diagnostic issues using the package ‘DHARMa’ (Hartig, 2019) with reference to the tests presented in the *plot* function (for normality, overdispersion, outliers, and homogeneity of variance). No model had significant diagnostic issues. All linear models (22–31: Table 1) were tested for homogeneity of variance and outliers, using the functions *check_homogeneity* and *check_outliers* from the package ‘performance’ (Lüdecke et al., 2021). There was no clear evidence for different variances across groups (Bartlett tests: P > 0.05). Where significant habitat-contrasts were found, any detected outliers (Cook’s distance: threshold = 0.5) were temporarily removed to ensure that they did not change the identification of significant contrasts (models 22 and 31).

The residuals of all parametric models (1–4 and 9–31: Table 1) were examined for spatial autocorrelation with correlograms using the *correlog* function in the package ‘ncf’ (Bjornstad, 2016). Spatial dependence was examined at discrete 1.5 km distance classes so as to encompass closely paired sites (ten out of the twelve) within the smallest distance class, and to provide sufficient pair-wise residual comparisons within each class for all analyses. Each paired understory and canopy trap (48 pairs in total) shared the same coordinates, so potential non-independence was examined separately within a ‘0 km’ class. There was no evidence of problematic spatial dependence in any model residuals, with no significant, positive Moran’s *I* values for models in which significant contrasts were identified, and none between 0 and 6 km for the remaining models.


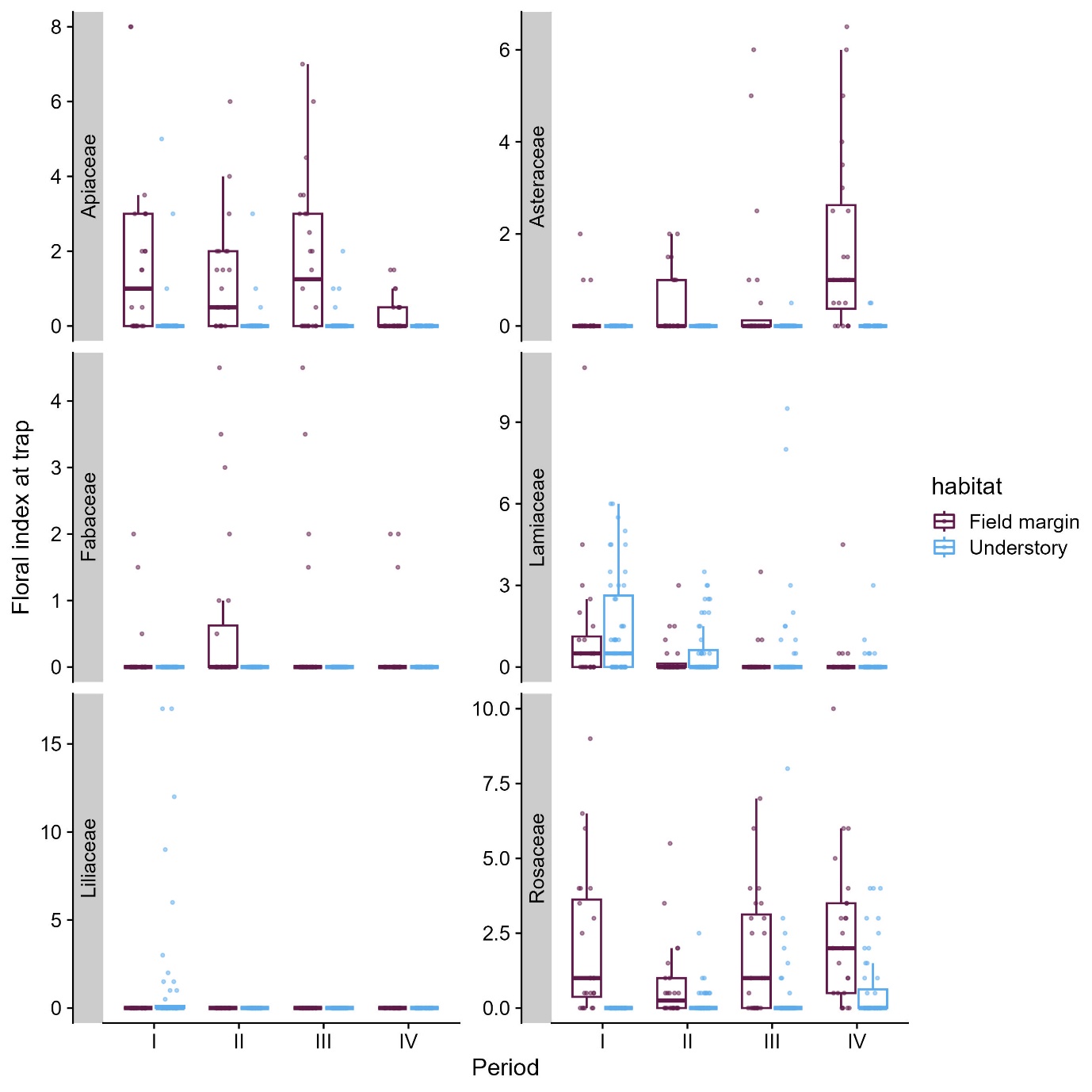


**Figure S1**. Floral abundance of the six most common plant families at each ground-level trap – see Appendix S1 for an explanation of floral index scores. Periods I, II, III, and IV represent the sampling periods in early May, late May, mid-June and mid-July, respectively. Note that ‘Liliaceae’ comprises *Hyacinthoides non-scripta* and *Allium ursinum*, which are now classified within Asparagaceae and Amaryllidaceae, respectively.


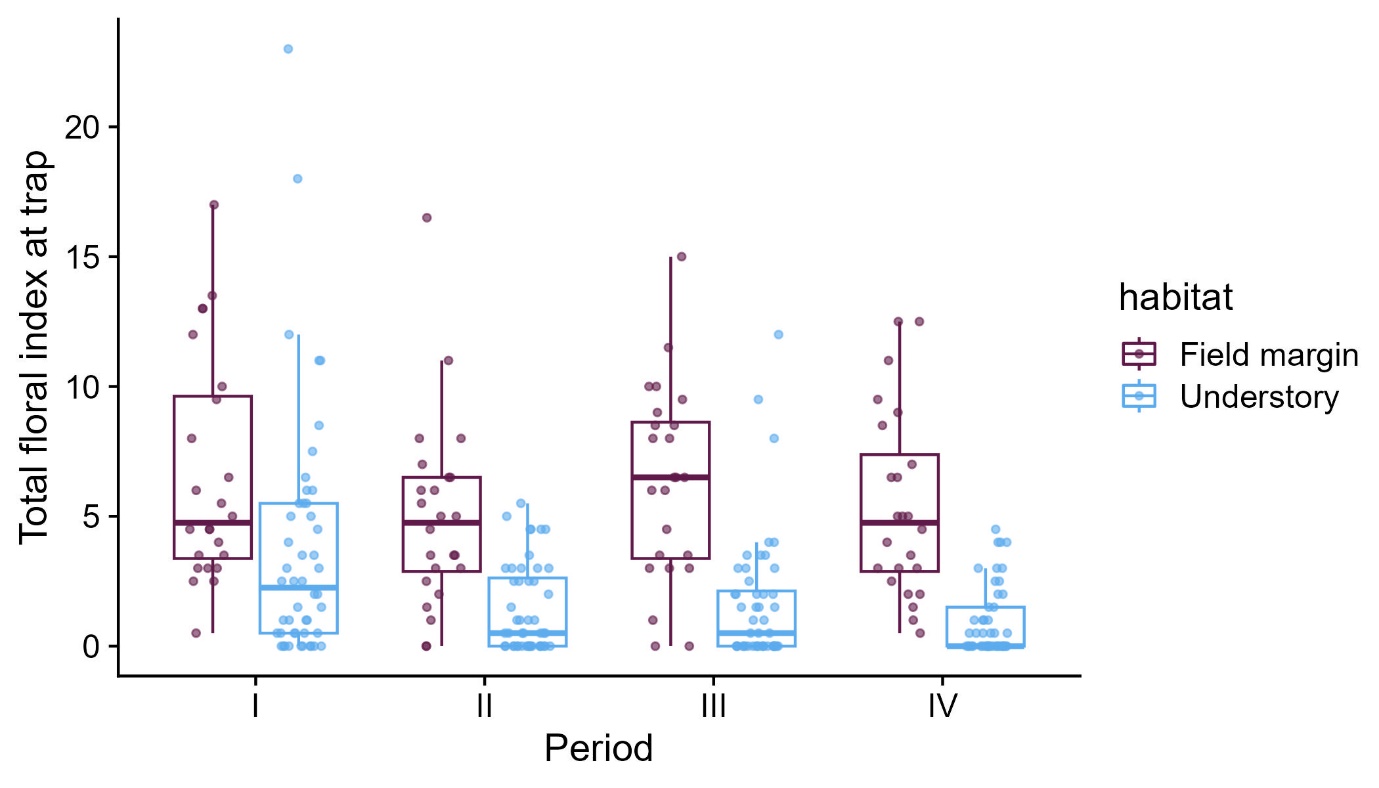
**Figure S2**. Combined floral abundance of all bee-visited plant families at each ground-level trap – see Appendix S1 for an explanation of floral index scores. Periods I, II, III, and IV represent the sampling periods in early May, late May, mid-June and mid-July, respectively.

**Table S1**. Species’ strength of understory association (mean of understory estimate minus field margin estimate across modelled periods [Table S2]) and eye parameter (taken from Tichit et al. (2024).

| **Species** | **Mean (Understory est. – Field margin est.) ± SE (n)** | **Eye parameter (µm.rad)** |
| --- | --- | --- |
| *B. lapidarius* | -2.64 ± 0.47 (4) | 0.69 |
| *B. terrestris* | -1.23 ± 0.35 (4) | 0.67 |
| *B. pascuorum* | -0.66 ± 0.38 (4) | 0.81 |
| *B. hypnorum* | -0.10 ± 0.04 (3) | 0.73 |
| *B. pratorum* | 0.17 ± 0.27 (4) | 0.82 |
| *B. hortorum* | 0.63 ± 0.19 (4) | 0.79 |

**Table S2.** Omnibus tests (likelihood ratio χ²) and pairwise contrasts of species trap-catch between habitats (models 1–4: Table 1). Omnibus test P values are adjusted using the Benjamini–Hochberg method for a family of nine test (models 1–8, plus the correlation test). ‘open’ = Field margin. Estimates are given on the log (not the response) scale. Contrast test P values are adjusted using the Tukey method for comparing a family of three estimates. P values < 0.05 are in **bold**.

| **Model (no.)** | **Likelihood ratio test** (*species* x *habitat*) | **contrast** | **species** | **estimate** | **SE** | **z.ratio** | **p.value** |
| --- | --- | --- | --- | --- | --- | --- | --- |
| **Early May (1)** | LRT = 40.4; **P < 0.001** | open - understory | *B. hortorum* | -0.454 | 0.532 | -0.854 | 0.670 |
|  |  | open - canopy |  | 1.648 | 0.849 | 1.941 | 0.127 |
|  |  | understory - canopy |  | 2.102 | 0.759 | 2.768 | **0.016** |
|  |  | open - understory | *B. hypnorum* | 0.016 | 0.878 | 0.018 | 1.000 |
|  |  | open - canopy |  | -0.655 | 0.804 | -0.815 | 0.694 |
|  |  | understory - canopy |  | -0.671 | 0.624 | -1.075 | 0.529 |
|  |  | open - understory | *B. lapidarius* | 1.536 | 0.475 | 3.233 | **0.004** |
|  |  | open - canopy |  | 0.732 | 0.382 | 1.916 | 0.134 |
|  |  | understory - canopy |  | -0.804 | 0.468 | -1.717 | 0.199 |
|  |  | open - understory | *B. pascuorum* | -0.146 | 0.323 | -0.454 | 0.893 |
|  |  | open - canopy |  | -0.073 | 0.325 | -0.224 | 0.973 |
|  |  | understory - canopy |  | 0.074 | 0.256 | 0.288 | 0.955 |
|  |  | open - understory | *B. pratorum* | -0.572 | 0.290 | -1.970 | 0.120 |
|  |  | open - canopy |  | 0.431 | 0.328 | 1.314 | 0.387 |
|  |  | understory - canopy |  | 1.003 | 0.255 | 3.936 | **0.000** |
|  |  | open - understory | *B. terrestris* | 0.257 | 0.504 | 0.510 | 0.866 |
|  |  | open - canopy |  | 1.068 | 0.603 | 1.771 | 0.179 |
|  |  | understory - canopy |  | 0.811 | 0.552 | 1.468 | 0.306 |
| **Late May (2)** | LRT = 84.2; **P < 0.001** | open - understory | *B. hortorum* | -0.364 | 0.286 | -1.273 | 0.411 |
|  |  | open - canopy |  | 1.476 | 0.351 | 4.208 | **0.000** |
|  |  | understory - canopy |  | 1.841 | 0.303 | 6.078 | **0.000** |
|  |  | open - understory | *B. hypnorum* | 0.126 | 0.367 | 0.345 | 0.936 |
|  |  | open - canopy |  | 0.701 | 0.395 | 1.774 | 0.178 |
|  |  | understory - canopy |  | 0.574 | 0.339 | 1.694 | 0.207 |
|  |  | open - understory | *B. lapidarius* | 2.345 | 0.499 | 4.697 | **0.000** |
|  |  | open - canopy |  | 1.777 | 0.418 | 4.251 | **0.000** |
|  |  | understory - canopy |  | -0.568 | 0.545 | -1.042 | 0.550 |
|  |  | open - understory | *B. pascuorum* | 0.753 | 0.322 | 2.341 | 0.050 |
|  |  | open - canopy |  | 1.544 | 0.364 | 4.240 | **0.000** |
|  |  | understory - canopy |  | 0.791 | 0.343 | 2.306 | 0.055 |
|  |  | open - understory | *B. pratorum* | 0.600 | 0.237 | 2.536 | **0.030** |
|  |  | open - canopy |  | 3.043 | 0.307 | 9.906 | **0.000** |
|  |  | understory - canopy |  | 2.442 | 0.279 | 8.742 | **0.000** |
|  |  | open - understory | *B. terrestris* | 1.948 | 0.378 | 5.147 | **0.000** |
|  |  | open - canopy |  | 2.471 | 0.430 | 5.741 | **0.000** |
|  |  | understory - canopy |  | 0.523 | 0.471 | 1.111 | 0.507 |

**Table S2** (*continued*).

| **Model (no.)** | **Likelihood ratio test**  (*species* x *habitat*) | **contrast** | **species** | **estimate** | **SE** | **z.ratio** | **p.value** |
| --- | --- | --- | --- | --- | --- | --- | --- |
| **Mid-June (3)** | LRT = 77.4; **P < 0.001** | open - understory | *B. hortorum* | -1.209 | 0.566 | -2.136 | 0.083 |
|  |  | open - canopy |  | 1.422 | 0.888 | 1.602 | 0.245 |
|  |  | understory - canopy |  | 2.631 | 0.750 | 3.509 | **0.001** |
|  |  | open - understory | *B. hypnorum* | 0.149 | 0.588 | 0.253 | 0.965 |
|  |  | open - canopy |  | 0.952 | 0.699 | 1.363 | 0.361 |
|  |  | understory - canopy |  | 0.803 | 0.623 | 1.290 | 0.401 |
|  |  | open - understory | *B. lapidarius* | 2.934 | 0.768 | 3.818 | **0.000** |
|  |  | open - canopy |  | 2.521 | 0.654 | 3.857 | **0.000** |
|  |  | understory - canopy |  | -0.413 | 0.927 | -0.446 | 0.896 |
|  |  | open - understory | *B. pascuorum* | 1.671 | 0.402 | 4.158 | **0.000** |
|  |  | open - canopy |  | 1.759 | 0.417 | 4.216 | **0.000** |
|  |  | understory - canopy |  | 0.088 | 0.466 | 0.188 | 0.981 |
|  |  | open - understory | *B. pratorum* | -0.237 | 0.275 | -0.862 | 0.664 |
|  |  | open - canopy |  | 2.464 | 0.484 | 5.088 | **0.000** |
|  |  | understory - canopy |  | 2.700 | 0.452 | 5.974 | **0.000** |
|  |  | open - understory | *B. terrestris* | 1.343 | 0.541 | 2.483 | **0.035** |
|  |  | open - canopy |  | 1.741 | 0.616 | 2.827 | **0.013** |
|  |  | understory - canopy |  | 0.398 | 0.666 | 0.598 | 0.821 |
| **Mid-July (4)** | LRT = 103.3; **P < 0.001** | open - understory | *B. hortorum* | -0.505 | 0.425 | -1.190 | 0.459 |
|  |  | open - canopy |  | 1.426 | 0.627 | 2.275 | 0.059 |
|  |  | understory - canopy |  | 1.931 | 0.548 | 3.522 | **0.001** |
|  |  | open - understory | *B. lapidarius* | 3.749 | 0.605 | 6.202 | **0.000** |
|  |  | open - canopy |  | 2.016 | 0.298 | 6.775 | **0.000** |
|  |  | understory - canopy |  | -1.733 | 0.633 | -2.737 | **0.017** |
|  |  | open - understory | *B. pascuorum* | 0.355 | 0.353 | 1.005 | 0.573 |
|  |  | open - canopy |  | 0.561 | 0.364 | 1.538 | 0.273 |
|  |  | understory - canopy |  | 0.205 | 0.332 | 0.619 | 0.810 |
|  |  | open - understory | *B. pratorum* | -0.472 | 0.453 | -1.041 | 0.551 |
|  |  | open - canopy |  | 1.292 | 0.641 | 2.017 | 0.108 |
|  |  | understory - canopy |  | 1.764 | 0.555 | 3.180 | **0.004** |
|  |  | open - understory | *B. terrestris* | 1.367 | 0.316 | 4.319 | **0.000** |
|  |  | open - canopy |  | 0.915 | 0.281 | 3.260 | **0.003** |
|  |  | understory - canopy |  | -0.452 | 0.318 | -1.422 | 0.329 |

**
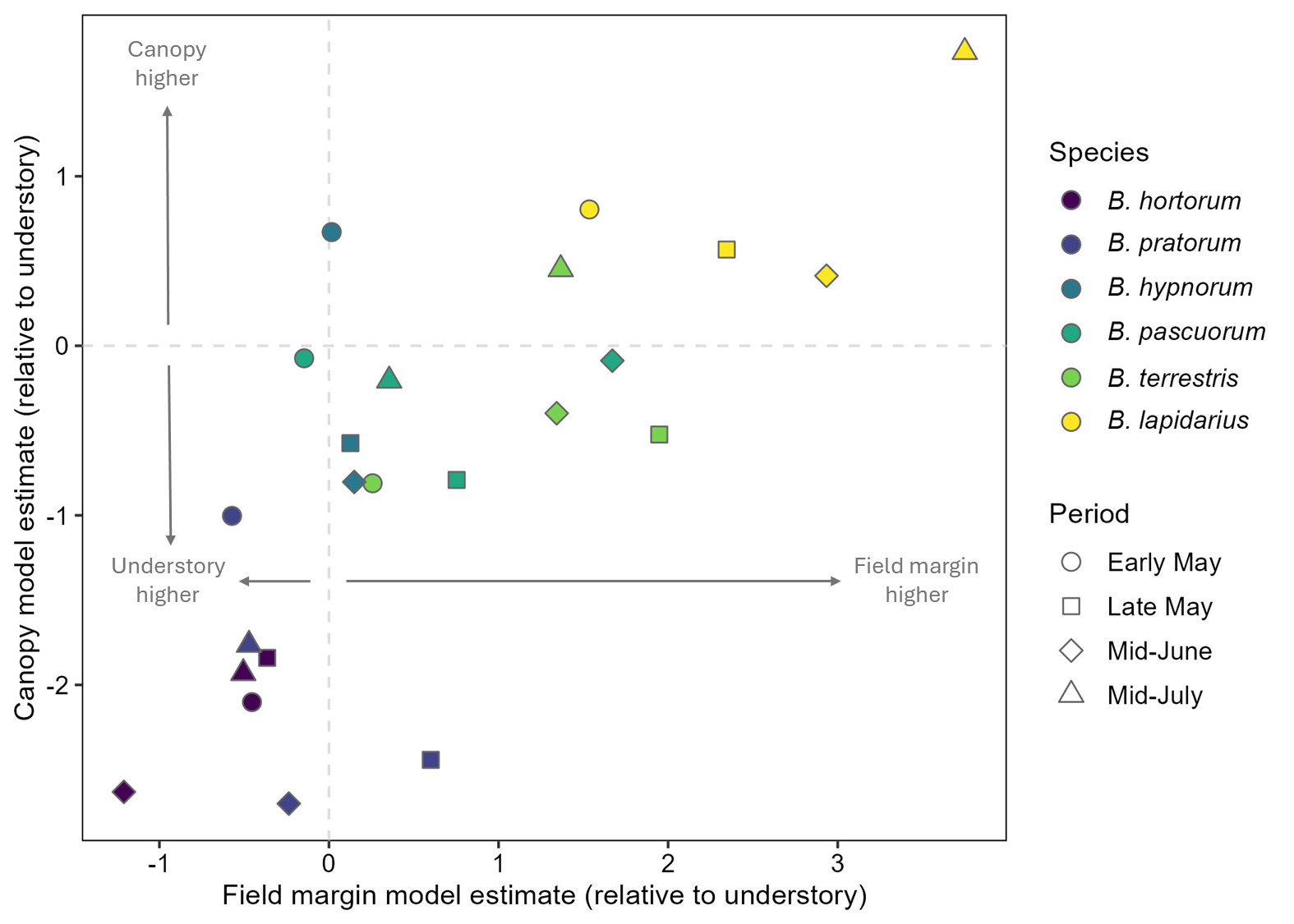
**

**Figure S3**. The relationship between field margin and canopy model-estimated mean bee abundance per trap (log scale), relative to the understory, across *Bombus* species and sampling periods (models 1–4).

**Table S3**. Omnibus tests (ANOVA) and pairwise contrasts of the ranked community-weighted means of eye parameter between habitats (models 5–8: Table 1). Omnibus test P values are adjusted using the Benjamini–Hochberg method for a family of nine tests (models 1–8, plus the correlation test). ‘open’ = Field margin. For mixed effects models (5, 7 and 8) degrees of freedom are estimated using the Kenward-Roger method. Contrast test P values are adjusted using the Tukey method for comparing a family of three estimates. P values < 0.05 are in **bold**.

| **Model (no.)** | **ANOVA test** (*habitat*) | **contrast** | **estimate** | **SE** | **df** | **t.ratio** | **p.value** |
| --- | --- | --- | --- | --- | --- | --- | --- |
| **Early May (5)** | F = 1.55; P = 0.234 | open - understory | -6.417 | 3.844 | 22.0 | -1.669 | 0.239 |
|  |  | open - canopy | -5.083 | 3.844 | 22.0 | -1.322 | 0.398 |
|  |  | understory - canopy | 1.333 | 3.844 | 22.0 | 0.347 | 0.936 |
| **Late May (6)** | F = 11.5; **P < 0.001** | open - understory | -8.958 | 3.398 | 33 | -2.636 | **0.033** |
|  |  | open - canopy | 7.333 | 3.398 | 33 | 2.158 | 0.094 |
|  |  | understory - canopy | 16.292 | 3.398 | 33 | 4.794 | **0.000** |
| **Mid-June (7)** | F = 6.53; **P = 0.008** | open - understory | -9.833 | 2.818 | 19.1 | -3.489 | **0.007** |
|  |  | open - canopy | -2.288 | 3.126 | 20.1 | -0.732 | 0.748 |
|  |  | understory - canopy | 7.545 | 3.126 | 20.1 | 2.414 | 0.063 |
| **Mid-July (8)** | F = 8.64; **P = 0.002** | open - understory | -13.458 | 3.282 | 22.0 | -4.100 | **0.001** |
|  |  | open - canopy | -4.792 | 3.282 | 22.0 | -1.460 | 0.329 |
|  |  | understory - canopy | 8.667 | 3.282 | 22.0 | 2.640 | **0.038** |

**Table S4**. Omnibus tests (likelihood ratio χ²) and pairwise contrasts of caste trap-catch between habitats (models 9–12: Table 1). Omnibus test P values are adjusted using the Benjamini–Hochberg method for a family of 13 tests (models 9–21). ‘open’ = Field margin. Estimates are given on the log (not the response) scale. Contrast test P values are adjusted using the Tukey method for comparing a family of three estimates. P values < 0.05 are in **bold**.

| **Model (no.)** | **Likelihood ratio test**  (*caste* x *habitat*) | **contrast** | **caste** | **estimate** | **SE** | **z.ratio** | **p.value** |
| --- | --- | --- | --- | --- | --- | --- | --- |
| **Early May (9)** | LRT = 1.29; P = 0.525 | open - understory | Worker | -0.188 | 0.256 | -0.732 | 0.745 |
|  |  | open - canopy |  | 0.195 | 0.268 | 0.728 | 0.747 |
|  |  | understory - canopy |  | 0.383 | 0.215 | 1.784 | 0.175 |
|  |  | open - understory | Reproductive | -0.011 | 0.248 | -0.046 | 0.999 |
|  |  | open - canopy |  | 0.554 | 0.268 | 2.067 | 0.097 |
|  |  | understory - canopy |  | 0.565 | 0.225 | 2.508 | **0.033** |
| **Late May (10)** | LRT = 113.3; **P < 0.001** | open - understory | Worker | 1.034 | 0.193 | 5.367 | **0.000** |
|  |  | open - canopy |  | 2.059 | 0.214 | 9.615 | **0.000** |
|  |  | understory - canopy |  | 1.025 | 0.193 | 5.307 | **0.000** |
|  |  | open - understory | Reproductive | -0.231 | 0.205 | -1.127 | 0.498 |
|  |  | open - canopy |  | 2.458 | 0.305 | 8.058 | **0.000** |
|  |  | understory - canopy |  | 2.689 | 0.281 | 9.582 | **0.000** |
| **Mid-June (11)** | LRT = 30.9; **P < 0.001** | open - understory | Worker | 0.708 | 0.233 | 3.036 | **0.007** |
|  |  | open - canopy |  | 2.186 | 0.304 | 7.183 | **0.000** |
|  |  | understory - canopy |  | 1.478 | 0.285 | 5.195 | **0.000** |
|  |  | open - understory | Reproductive | -1.381 | 0.469 | -2.942 | **0.009** |
|  |  | open - canopy |  | 0.436 | 0.573 | 0.761 | 0.727 |
|  |  | understory - canopy |  | 1.817 | 0.413 | 4.403 | **0.000** |
| **Mid-July (12)** | LRT = 33.3; **P < 0.001** | open - understory | Worker | 1.585 | 0.218 | 7.273 | **0.000** |
|  |  | open - canopy |  | 1.404 | 0.208 | 6.738 | **0.000** |
|  |  | understory - canopy |  | -0.181 | 0.228 | -0.796 | 0.706 |
|  |  | open - understory | Reproductive | -0.043 | 0.270 | -0.159 | 0.986 |
|  |  | open - canopy |  | 0.943 | 0.320 | 2.943 | **0.009** |
|  |  | understory - canopy |  | 0.986 | 0.279 | 3.534 | **0.001** |

**Table S5**. Omnibus tests (likelihood ratio χ²) and pairwise contrasts of the log odds ratio of reproductive (male and/or queen) presence between habitats (models 13–21: Table 1). Omnibus test P values are adjusted using the Benjamini–Hochberg method for a family of 13 tests (models 9–21). ‘open’ = Field margin. Estimates are averaged over levels of *site* where this is included as a fixed factor (models 17, 18, 20). Where three habitats are included, contrast test P values are adjusted using the Tukey method. P values < 0.05 are in **bold**.

| **Model (no.)** | **Likelihood ratio test**  (*habitat*) | **contrast** | **estimate** | **SE** | **z.ratio** | **p.value** |
| --- | --- | --- | --- | --- | --- | --- |
| **Early May;  *B. pascuorum* (13)** | LRT = 16.7; **P < 0.001** | open - understory | -1.925 | 1.037 | -1.856 | 0.152 |
|  |  | open - canopy | 1.235 | 0.862 | 1.432 | 0.324 |
|  |  | understory - canopy | 3.160 | 0.920 | 3.434 | **0.002** |
| **Early May;  *B. pratorum* (14)** | LRT = 3.09; P = 0.275 | open - understory | -1.672 | 1.136 | -1.472 | 0.305 |
|  |  | open - canopy | -1.769 | 1.340 | -1.320 | 0.384 |
|  |  | understory - canopy | -0.097 | 0.835 | -0.116 | 0.993 |
| **Late May; *B. pascuorum* (15)** | LRT = 8.51; **P = 0.023** | open - understory | -0.875 | 0.501 | -1.745 | 0.189 |
|  |  | open - canopy | 1.477 | 1.093 | 1.352 | 0.367 |
|  |  | understory - canopy | 2.351 | 1.081 | 2.175 | 0.075 |
| **Late May; *B. pratorum* (16)** | LRT = 54.2; **P < 0.001** | open - understory | -1.390 | 0.196 | -7.081 | **0.000** |
|  |  | open - canopy | -0.884 | 0.453 | -1.951 | 0.125 |
|  |  | understory - canopy | 0.506 | 0.444 | 1.139 | 0.490 |
| **Late May;  *B. hypnorum* (17)** | LRT = 2.91; P = 0.275 | open - understory | -1.109 | 0.819 | -1.354 | 0.365 |
|  |  | open - canopy | 0.410 | 1.044 | 0.393 | 0.918 |
|  |  | understory - canopy | 1.519 | 1.064 | 1.428 | 0.326 |
| **Late May;  *B. hortorum* (18)** | LRT = 27.5; **P < 0.001** | open - understory | -1.551 | 0.449 | -3.455 | **0.002** |
|  |  | open - canopy | 1.604 | 0.819 | 1.958 | 0.123 |
|  |  | understory - canopy | 3.155 | 0.797 | 3.960 | **0.000** |
| **Mid-June;  *B. pratorum* (19)** | LRT = 2.29; P = 0.188 | open - understory | -0.985 | 0.686 | -1.436 | 0.151 |
| **Mid-July;  *B. lapidarius* (20)** | LRT = 1.22; P = 0.291 | open - canopy | -0.884 | 0.788 | -1.122 | 0.262 |
| **Mid-July;  *B. terrestris* (21)** | LRT = 13.5; **P = 0.002** | open - understory | -2.303 | 0.671 | -3.433 | **0.002** |
|  |  | open - canopy | -1.063 | 0.586 | -1.813 | 0.165 |
|  |  | understory - canopy | 1.240 | 0.627 | 1.976 | 0.118 |

**Table S6**. Summary of bumblebee counts comprising the analyses presented in Figure 5. Reproductive and worker castes are in **bold**. F = Field margin; U = understory; C = Canopy.

| Figure panel | Sampling period | Species | Habitats modelled | Males | Queens | Total reproductives | Workers | Total bees | Sample size range |
| --- | --- | --- | --- | --- | --- | --- | --- | --- | --- |
| a | Early May | *B. pascuorum* | F, U, C | 0 | 75 | **75** | **20** | 95 | 1 – 10 |
| b | Early May | *B. pratorum* | F, U, C | 1 | 15 | **16** | **103** | 119 | 1 – 13 |
| c | Late May | *B. pascuorum* | F, U, C | 1 | 23 | **24** | **79** | 103 | 1 – 11 |
| d | Late May | *B. pratorum* | F, U, C | 203 | 8 | **211** | **403** | 614 | 1 – 75 |
| e | Late May | *B. hypnorum* | F, U, C | 15 | 3 | **18** | **59** | 77 | 1 – 9 |
| f | Late May | *B. hortorum* | F, U, C | 98 | 14 | **112** | **87** | 199 | 1 – 21 |
| g | Mid-June | *B. pratorum* | F, U | 15 | 8 | **23** | **101** | 124 | 1 – 21 |
| h | Mid-July | *B. lapidarius* | F, C | 13 | 4 | **17** | **66** | 83 | 1 – 17 |
| i | Mid-July | *B. terrestris* | F, U, C | 26 | 3 | **29** | **55** | 84 | 1 – 7 |

**Table S7**. Total bumblebees sampled in each habitat according to species and period, with the percentage comprising reproductives (queens and/or males) in parentheses. Understory percentages are in **bold** and/or *italics* if they are larger than the field margin and canopy percentages, respectively. Only the six most abundant social bumblebee species are displayed.

| Period | Species | Field margin | Understory | Canopy |
| --- | --- | --- | --- | --- |
| Early May | *B. hortorum* | 5 (20.0) | 16 (***25.0***) | 2 (0.0) |
|  | *B. hypnorum* | 2 (0.0) | 4 (***50.0***) | 8 (0.0) |
|  | *B. lapidarius* | 16 (93.8) | 7 (***100.0***) | 16 (68.8) |
|  | *B. pascuorum* | 17(76.5) | 40 (***95.0***) | 38 (63.2) |
|  | *B. pratorum* | 20 (5.0) | 72 (***16.7***) | 27 (11.1) |
|  | *B. terrestris* | 7(85.7) | 11 (**100.0**) | 5 (100.0) |
| Late May | *B. hortorum* | 54 (35.2) | 123 (***73.2***) | 22 (13.6) |
|  | *B. hypnorum* | 21(28.6) | 35 (*28.6*) | 21 (9.5) |
|  | *B. lapidarius* | 53 (20.8) | 6 (***50.0***) | 11 (9.1) |
|  | *B. pascuorum* | 46 (19.6) | 38 (***36.8***) | 19 (5.3) |
|  | *B. pratorum* | 297 (20.2) | 291 (***48.8***) | 26 (34.6) |
|  | *B. terrestris* | 68 (32.4) | 14 (***42.9***) | 9 (22.2) |
| Mid-June | *B. hortorum* | 4 (50.0) | 28 (***64.3***) | 2 (50.0) |
|  | *B. hypnorum* | 5 (0.0) | 9 (***33.3***) | 4 (0.0) |
|  | *B. lapidarius* | 18 (0.0) | 2 (***50.0***) | 3 (0.0) |
|  | *B. pascuorum* | 28 (3.6) | 11 (***45.5***) | 10 (40.0) |
|  | *B. pratorum* | 34 (8.8) | 90 (***22.2***) | 6 (0.0) |
|  | *B. terrestris* | 11 (0.0) | 6 (**33.3**) | 4 (50.0) |
| Mid-July | *B. hortorum* | 8 (20.0) | 26 (***96.2***) | 4 (25.0) |
|  | *B. hypnorum* | 0 | 4 (50.0) | 2 (100.0) |
|  | *B. lapidarius* | 65 (20.0) | 3 (***66.7***) | 18 (22.2) |
|  | *B. pascuorum* | 16 (6.3) | 22 (***18.2***) | 19 (5.3) |
|  | *B. pratorum* | 7 (14.3) | 22 (**27.3**) | 4 (50.0) |
|  | *B. terrestris* | 36(16.7) | 18 (***66.7***) | 30 (36.7) |

**Table S8**. Omnibus tests (ANOVA) and pairwise contrasts of worker intertegular-distance between habitats (models 22–31: Table 1). Omnibus test P values are adjusted using the Benjamini–Hochberg method for a family of ten tests. ‘field’ = Field margin. For mixed effects models (22–24, 26–29, 31), degrees of freedom are estimated using the Kenward-Roger method. Where three habitats are included, contrast test P values are adjusted using the Tukey method. P values < 0.05 are in **bold**.

| **Model (no.)** | **Anova test** (*habitat*) | **contrast** | **estimate** | **SE** | **df** | **t.ratio** | **p.value** |
| --- | --- | --- | --- | --- | --- | --- | --- |
| **Early May;  *B. pratorum* (22)** | F = 3.97;  P = 0.083 | field - understory | -0.176 | 0.063 | 51.3 | -2.774 | **0.021** |
|  |  | field - canopy | -0.159 | 0.074 | 56.0 | -2.148 | 0.089 |
|  |  | understory - canopy | 0.017 | 0.059 | 51.5 | 0.285 | 0.956 |
| **Late May;  *B. pratorum* (23)** | F = 0.51;  P = 0.750 | field - understory | -0.014 | 0.033 | 38.4 | -0.428 | 0.904 |
|  |  | field - canopy | 0.048 | 0.063 | 180.8 | 0.765 | 0.725 |
|  |  | understory - canopy | 0.062 | 0.062 | 230.0 | 0.999 | 0.578 |
| **Late May;  *B. pascuorum* (24)** | F = 5.33;  P = 0.058 | field - understory | -0.175 | 0.074 | 19.7 | -2.358 | 0.071 |
|  |  | field - canopy | -0.246 | 0.083 | 30.0 | -2.965 | **0.016** |
|  |  | understory - canopy | -0.071 | 0.089 | 39.4 | -0.799 | 0.706 |
| **Late May;  *B.lapidarius* (25)** | F = 0.43;  P = 0.733 | field - canopy | 0.061 | 0.093 | 50 | 0.658 | 0.513 |
| **Late May;  *B. hypnorum* (26)** | F =0.77;  P = 0.733 | field - understory | 0.053 | 0.083 | 31.0 | 0.639 | 0.800 |
|  |  | field - canopy | -0.042 | 0.087 | 31.8 | -0.484 | 0.879 |
|  |  | understory - canopy | -0.095 | 0.077 | 28.4 | -1.234 | 0.443 |
| **Late May;  *B. hortorum* (27)** | F = 0.81;  P = 0.733 | field - understory | -0.129 | 0.119 | 34.9 | -1.080 | 0.533 |
|  |  | field - canopy | -0.151 | 0.136 | 36.2 | -1.117 | 0.510 |
|  |  | understory - canopy | -0.022 | 0.129 | 39.0 | -0.174 | 0.983 |
| **Mid-June;  *B. pratorum* (28)** | F = 0.05; P = 0.818 | field - understory | -0.016 | 0.071 | 37.3 | -0.231 | 0.818 |
| **Mid-July; *B. pascuorum* (29)** | F = 1.96; P = 0.404 | field - understory | -0.134 | 0.117 | 25.1 | -1.140 | 0.499 |
|  |  | field - canopy | -0.234 | 0.118 | 21.2 | -1.981 | 0.141 |
|  |  | understory - canopy | -0.100 | 0.104 | 27.3 | -0.961 | 0.607 |
| **Mid-July;  *B. lapidarius* (30)** | F = 0.07; P = 0.818 | field - canopy | -0.023 | 0.085 | 64 | -0.273 | 0.786 |
| **Mid-July;  *B. terrestris* (31)** | F = 7.35; P = 0.058 | field - canopy | -0.327 | 0.121 | 26.3 | -2.712 | **0.012** |

**Table S9**. Summary of social bumblebee caste counts in each sampling period (with percentages in parentheses)

| Social *Bombus* caste | Sampling period | | | |
| --- | --- | --- | --- | --- |
|  | Early May | Late May | Mid-June | Mid-July |
| queens | 148 (**47**) | 83 (**7.1**) | 20 (**7.2**) | 19 (**6.1**) |
| males | 5 (**2.0**) | 330 (**28**) | 44 (**16**) | 84 (**27**) |
| workers | 160 (**51**) | 752 (**65**) | 215 (**77**) | 207 (**67**) |
| Total | 313 (**100**) | 1165 (**100**) | 279 (**100**) | 310 (**100**) |

**Appendix S3.** Post hoc testing of *floral index*.

Allen et al. (2025) found no relationship between floral resources (*floral index*) and either field margin or understory trap catches of total bees. They concluded that the distracting effect of flowers in the vicinity of traps may preclude such relationships from occurring (as discussed in Mathis et al., 2024). To confirm its lack of influence in the present study, we added *floral index*, post hoc, to models of species and caste trap-catch (1–4 and 9–12). First, we included *floral index* in a three-way interaction with *habitat* and *species*/*caste*, and second, we included it as a separate additive term. We then repeated this process on datasets that exclude canopy traps, as ground-level floral abundance may not be expected to predict catches of traps that are 13.5–20 m above ground. In all 32 model reruns, the three-way interactions, and *floral index* as an additive term, were non-significant (likelihood ratio χ² tests). Results are presented in the table below. P values have not been adjusted to correct for multiple testing.

| **Model no.** | **Traps excluded** | **Model term** | **Likelihood ratio test** | **P value** |
| --- | --- | --- | --- | --- |
| 1 | None | *species***habitat***floral index* | 10.19 | 0.424 |
|  | None | *floral index* | 0.08 | 0.780 |
|  | Canopy | *species***habitat***floral index* | 3.73 | 0.589 |
|  | Canopy | *floral index* | 1.81 | 0.179 |
| 2 | None | *species***habitat***floral index* | 12.39 | 0.260 |
|  | None | *floral index* | 0.09 | 0.767 |
|  | Canopy | *species***habitat***floral index* | 6.42 | 0.268 |
|  | Canopy | *floral index* | 0.64 | 0.425 |
| 3 | None | *species***habitat***floral index* | 9.50 | 0.485 |
|  | None | *floral index* | 0.02 | 0.901 |
|  | Canopy | *species***habitat***floral index* | 7.30 | 0.200 |
|  | Canopy | *floral index* | 0.37 | 0.545 |
| 4 | None | *species***habitat***floral index* | 12.39 | 0.135 |
|  | None | *floral index* | 0.27 | 0.601 |
|  | Canopy | *species***habitat***floral index* | 9.34 | 0.053 |
|  | Canopy | *floral index* | 1.41 | 0.235 |
| 9 | None | *caste***habitat***floral index* | 0.47 | 0.790 |
|  | None | *floral index* | 0.08 | 0.780 |
|  | Canopy | *caste***habitat***floral index* | 0.39 | 0.533 |
|  | Canopy | *floral index* | 1.81 | 0.179 |
| 10 | None | *caste***habitat***floral index* | 2.85 | 0.240 |
|  | None | *floral index* | 0.20 | 0.655 |
|  | Canopy | *caste***habitat***floral index* | 2.74 | 0.098 |
|  | Canopy | *floral index* | 0.93 | 0.335 |
| 11 | None | *caste***habitat***floral index* | 0.39 | 0.823 |
|  | None | *floral index* | 0.01 | 0.933 |
|  | Canopy | *caste***habitat***floral index* | 0.01 | 0.921 |
|  | Canopy | *floral index* | 0.36 | 0.551 |
| 12 | None | *caste***habitat***floral index* | 0.39 | 0.824 |
|  | None | *floral index* | 0.44 | 0.509 |
|  | Canopy | *caste***habitat***floral index* | 0.33 | 0.563 |
|  | Canopy | *floral index* | 1.65 | 0.199 |

**References**

Allen, G., Dicks, L.V., Taylor, M.I., Hewitt, D. & Davies, R.G. (2025). Localised patterns of wild bee abundance indicate woodlands play multiple roles in supporting farmland populations. *Insect Conservation and Diversity*. 18, 997-1012.

Bjornstad, O. N. (2016) ncf: spatial covariance functions. R package version 1.3-2. <https://CRAN.R-project.org/package=ncf>

DoPI (The Database of Pollinator Interactions) (2022) <https://www.dopi.org.uk/search> [Accessed April 2022].

Hartig, F. (2019) DHARMa: residual diagnostics for hierarchical (multi-level/mixed) regression models. R package version 0.4.7. <https://CRAN.R-project.org/package=DHARMa>.

Mathis, C.L., McNeil, D.J., Jr., Kammerer, M., Larkin, J.L. & Skvarla, M.J. (2024) Distance models reveal biases associated with passive trapping methods for measuring wild bee abundance. Frontiers in Ecology and Evolution, 12, 1380622.

Tichit, P., Kendall, L., Olsson, P., Taylor, G., Rau, C., Caplat, P., ... & Baird, E. (2024). The Interplay Between Visual Traits and Forest in Bumblebee Communities Across Sweden. *Ecology and Evolution*, *14*(12), e70635.

Wood, T. J., Ghisbain, G., Rasmont, P., Kleijn, D., Raemakers, I., Praz, C., ... & Michez, D. (2021) Global patterns in bumble bee pollen collection show phylogenetic conservation of diet. *Journal of Animal Ecology*, 90, 2421-2430.
